# Frequency of coexistent eye diseases and cognitive impairment or dementia: a systematic review and meta-analysis

**DOI:** 10.1038/s41433-023-02481-4

**Published:** 2023-03-15

**Authors:** Ying Xu, Jack Phu, Htein Linn Aung, Negin Hesam-Shariati, Lisa Keay, Phillip J. Tully, Andrew Booth, Craig S. Anderson, Kaarin J. Anstey, Ruth Peters

**Affiliations:** 1 Neuroscience Research Australia, Sydney, NSW Australia; 2 School of Psychology, Faculty of Science, UNSW, Sydney, NSW Australia; 3 The George Institute for Global Health, Faculty of Medicine, UNSW, Sydney, NSW Australia; 4 Faculty of Medicine, UNSW, Sydney, NSW Australia; 5 Ageing Futures Institute, UNSW, Sydney, NSW Australia; 6 Centre for Eye Health, UNSW, Sydney, NSW Australia; 7 School of Optometry and Vision Science, UNSW, Sydney, NSW Australia; 8 Faculty of Medicine and Health, University of Sydney, Sydney, NSW Australia; 9 Concord Clinical School, Concord Repatriation General Hospital, Sydney, NSW Australia; 10 School of Psychology, The University of New England, Armidale, NSW Australia; 11 School of Health and Related Research, University of Sheffield, Sheffield, UK; 12 The George Institute for Global Health, Beijing, P.R. China; 13 Neurology Department, Royal Prince Alfred Hospital, Sydney Local Area Health District, Sydney, NSW Australia; 14 School of Public Health, Imperial College London, London, UK

**Keywords:** Retinal diseases, Predictive markers

## Abstract

**Objective:**

We aim to quantify the co-existence of age-related macular degeneration (AMD), glaucoma, or diabetic retinopathy (DR) and cognitive impairment or dementia.

**Method:**

MEDLINE, EMBASE, PsycINFO and CINAHL were searched (to June 2020). Observational studies reporting incidence or prevalence of AMD, glaucoma, or DR in people with cognitive impairment or dementia, and of cognitive impairment or dementia among people with AMD, glaucoma, or DR were included.

**Results:**

Fifty-six studies (57 reports) were included but marked by heterogeneities in the diagnostic criteria or definitions of the diseases, study design, and case mix. Few studies reported on the incidence. Evidence was sparse but consistent in individuals with mild cognitive impairment where 7.7% glaucoma prevalence was observed. Prevalence of AMD and DR among people with cognitive impairment ranged from 3.9% to 9.4% and from 11.4% to 70.1%, respectively. Prevalence of AMD and glaucoma among people with dementia ranged from 1.4 to 53% and from 0.2% to 25.9%, respectively. Prevalence of DR among people with dementia was 11%. Prevalence of cognitive impairment in people with AMD, glaucoma, and DR ranged from 8.4% to 52.4%, 12.3% to 90.2%, and 3.9% to 77.8%, respectively, and prevalence of dementia in people with AMD, glaucoma and DR ranged from 9.9% to 62.6%, 2.5% to 3.3% and was 12.5%, respectively.

**Conclusions:**

Frequency of comorbid eye disease and cognitive impairment or dementia varied considerably. While more population-based estimations of the co-existence are needed, interdisciplinary collaboration might be helpful in the management of these conditions to meet healthcare needs of an ageing population.

**Trial registration:**

PROSPERO registration: CRD42020189484.

## Abstract (zh)

目的: 我们旨在量化老年黄斑变性 (AMD) 、青光眼或糖尿病视网膜疾病合并认知障碍或痴呆情况。

方法: 本文检索了MEDLINE、EMBASE、PsycINFO和CINAHL (至2020年6月) 数据库。观察性研究报道了认知障碍或痴呆患者中AMD、青光眼或糖尿病视网膜病变 (DR) 的发病率或流行率, 以及AMD、青光眼或DR患者中认知障碍或痴呆的情况。

结果: 本文共纳入了56项研究 (57份报告), 这些研究在疾病的诊断标准或定义、研究设计和病例组合方面存在着异质性。很少研究报告发病率。虽然证据少, 但在轻度认知障碍者中的证据是连续的, 观察到7.7%的青光眼发病率。认知障碍者中AMD和DR的患病率分别为3.9%至9.4%和11.4%至70.1%。痴呆症患者中AMD和青光眼的患病率分别为1.4%至53%和0.2%至25.9%。痴呆症患者中DR的患病率为11%。

患有AMD、青光眼和DR的人中认知障碍的发病率分别为8.4%至52.4%、12.3%至90.2%和3.9%至77.8%, 患有AMD、青光眼和DR的人中痴呆症的发病率分别为9.9%至62.6%、2.5%至3.3%和12.5%。

结论: 眼病合并认知障碍或痴呆的频率差异性大。虽然需要更多基于人口的共病估计, 但跨学科合作可能有助于管理这些疾病以满足老龄化人口的医疗保健需求。

## Introduction

Dementia affects around 50 million people, and there are nearly 10 million new cases each year worldwide [1]. Among general populations (aged ≥60 years), the prevalence of dementia has been estimated as ranging from 4.6% in Central Europe to 8.7% in North Africa and the Middle East [2], and prevalence estimates are inevitably influenced by diagnostic criteria and information source (e.g., claims or pharmacy data, or survey) [3]. Mild cognitive impairment (MCI) is a diagnosis that refers to impaired cognition [4], which is not severe enough to interfere with independent daily functioning. MCI is not a static condition with some cases progressing to dementia and some reverting to normal cognitive function [4]. Furthermore, concerns about the heterogeneity in the diagnostic and operational criteria for MCI have also been raised [4], and MCI has been applied to cognitive impairment no dementia, age-associated memory impairment, MCI, and amnestic MCI [4]. Likely due to this heterogeneity and the age of the studied participants, there has been substantial disparity in the reported prevalence of MCI, ranging from 0.5 to 42% [4].

The retina, being an extension to the brain, is particularly relevant as a potential window to explore dementia, because of the retinal-brain embryological, anatomical, and physiological connections. Thinning of the retinal nerve fibre layer and ganglion cell inner plexiform layer measured through optical coherence tomography retina scans has been reported in individuals with MCI and Alzheimer’s disease (AD) [5, 6]. Retinal microvascular variations have been investigated as biomarkers for cerebral microvasculature changes [7], and sparser retinal microvascular networks, increased tortuosity in the retinal vessels, and reduced blood flow are associated with cognitive decline, MCI, and dementia [8–10].

Eye diseases that affect the retina including age-related macular degeneration (AMD), glaucoma and diabetic retinopathy (DR) are leading causes of blindness among adults [11–13]. Meta-analyses of 39 and 50 population-based studies, and of individual participant data from 35 studies conducted in the USA, Europe, Asia and Australia, estimated that globally 170 million (in 2014, pooled prevalence of 8.7%), 64 million (in 2013, 3.5% of population aged 40 to 80 years), and 93 million (in 2010, 34.6% of those with diabetes) people had AMD, glaucoma and DR, respectively [11–13].

There are commonalities between AMD, glaucoma, or DR, and cognitive impairment or dementia. Clinically, they are all chronic and progressive, with strong family history connections and increasing incidence with age [14–16]. Pathophysiologically, common characteristics for AMD and AD include the presence of amyloid β in the drusen of AMD patients [14]. Amyloid β has been suggested as a target for glaucoma treatment [17] and other shared characteristics between glaucoma and dementia include common gene coding *OPTINEURIN* [18], neuroinflammation [19], and elevated levels of tumour necrosis factor α [20]. Common risk factors for DR, brain amyloid burden, and cognitive impairment include hyperglycaemia and insulin resistance [21].

To our knowledge, there is no existing systematic review evidence on the frequency of these eye diseases in people with cognitive impairment or dementia, or the frequency of cognitive impairment or dementia in people with these eye diseases. Knowledge of these relationships can help promote timely referral and inter-disciplinary communications between healthcare professionals, e.g., optometrists, ophthalmologists, diabetologists, geriatricians and neurologists, involved in the management of such co-morbidities.

## Methods

The protocol was registered in PROSPERO (CRD42020189484). Reporting of the review was followed the Meta-analysis Of Observational Studies in Epidemiology (MOOSE) guideline and Preferred Reporting Items for Systematic Reviews and Meta-Analyses (PRISMA) checklist.

### Inclusion and exclusion criteria

The population of interest included studies reporting on participants (aged ≥18 years) with any types and stages of AMD, glaucoma, DR, cognitive impairment, or dementia. Studies specifically on people with comorbid AMD, glaucoma or DR and cognitive impairment or dementia in the context of other diseases were excluded, except for those on comorbid DR and cognitive impairment or dementia in people with diabetes since DR is a complication of diabetes.

Published observational studies with consecutive, random, or convenience sampling were eligible. Studies with selective sampling were excluded. Observational study designs were accepted except for case-reports, case series, and studies of fewer than 50 participants. Outcomes were incidence and prevalence of AMD, glaucoma, or DR, in people with cognitive impairment or dementia, and/or incidence and prevalence of cognitive impairment or dementia in people with AMD, glaucoma or DR.

### Search strategy and screening

Four databases were searched: MEDLINE, EMBASE, PsycINFO and CINAHL (from inception to 1 June 2020, eTable 1). The following search terms were used as free text or controlled vocabulary as appropriate for the corresponding database: macular degeneration, geographic atrophy or dystrophy, macular oedema, vitelliform macular dystrophy, retina degeneration, drusen, glaucoma, ocular or eye hypertension, diabetic retinopathy AND Alzheimer(s), dementia, cognitive dysfunction.

No language or publication date restriction was applied. All identified titles and abstracts were screened by at least one of three reviewers, and 20% of the titles and abstracts were screened independently by two of the three reviewers. Full texts of relevant documents were obtained and independently assessed for relevance by two reviewers. All differences were resolved by discussion with the lead reviewer until the final set was agreed. Reference lists or citation trails of the included studies were checked to identify additional studies.

### Statistical analyses

To estimate frequencies, within study variances were calculated as square root of (*p*  ×  (1 − p)/n), where p is the cumulative incidence or prevalence, and n is the sample size. The cumulative incidence and prevalence rates were sorted from lowest to highest rates and plotted with 95% confidence intervals (CIs). Population-based studies were listed first in forest plots, easing visual comparisons to the other studies. Statistical heterogeneity and consistency were assessed using the standard I^2^ and Q statistic, with *p* < 0.05 indicating heterogeneity and I^2^ > 75% indicating high inconsistency. Rates were synthesized using a random effect inverse variance approach for weighting. We also conducted meta-analyses to provide summary estimates of the associations between each eye disease and cognitive impairment or dementia, based on multivariable adjusted odds ratios (ORs), relative risks (RRs), or hazard ratios (HRs). All analyses were conducted using Stata 13.

### Data extraction and risk of bias assessment

Data extraction was completed by two reviewers independently and compared and compiled by the lead reviewer. Risk of bias was assessed using the risk of bias tool for prevalence studies developed by Hoy et al (Appendix A) [22]. There were 9 items, and each item was judged as “low” versus “high risk of bias”.

## Results

The search results and selection process are summarised in a PRISMA flowchart (eFig. 1). After removing duplicates, a total of 5496 titles and abstracts were screened, including 1098 (20%) screened independently by two reviewers. Discrepancies occurred on 21 (1.9%) records of titles and abstracts, and were solved through discussion. Seventy-four full text reports were retrieved and assessed for inclusion/exclusion. Twenty-nine reports were excluded with reasons (Appendix B) and a total of 56 studies (57 reports, two reports on the same study [23, 24], Appendix C) were considered eligible for inclusion.

Twenty-four studies (25 reports) reported AMD, glaucoma or DR among people with cognitive impairment or dementia [23–47], and 34 studies reported cognitive impairment or dementia among people with AMD, glaucoma or DR [41, 43, 48–79]. These included two longitudinal studies [41, 43] reporting on both directions of the association: (1) cumulative incidence of AMD among people with dementia and cumulative incidence of dementia among AMD [41]; and (2) cumulative incidence of POAG among people with dementia and cumulative incidence of dementia among POAG [43]. Studies used four methodologies for eye diseases (Appendix D): retinal photograph grading, clinical examination, diagnostic coding, and histopathological assessments. For cognition, studies used standard diagnostic criteria for MCI or cut-offs on cognitive screening tools. Dementia was mainly assessed according to diagnostic criteria or diagnostic coding.

### Eye diseases among people with cognitive impairment or dementia

Studies were conducted in Australia [25, 26], Canada [27], China [28], France [29], Germany [30, 47], Greece [31], Italy [32], Japan [33, 34], the Philippines [35], Poland [23, 24], Singapore [36], Spain [37], Taiwan [38–40], the UK [41–44] and the USA [45, 46], and published between 1986 and 2020 (eTable 2). Twenty studies (83.3% of the studies, 21 reports) were published in the decade since 2011. There were nine population-based [25, 36, 38–41, 43–45], three community-based [29, 37, 47], 11 (12 reports) hospital-based [23, 24, 26–28, 31–35, 42, 46], and one nursing home based [30] studies. Sixteen studies (17 reports) were cross-sectional, and eight studies were longitudinal with varied follow up times reported as a mean and standard deviation of 7.6 ± 1 [25] and 2 ± 0.4 years [30] of follow-up, at least one year of follow-up [41, 43, 47], or reporting that AMD or glaucoma was diagnosed before the diagnosis of dementia (length of follow-up not reported) [38–40].

#### Among people with cognitive impairment

**Fig. 1:**
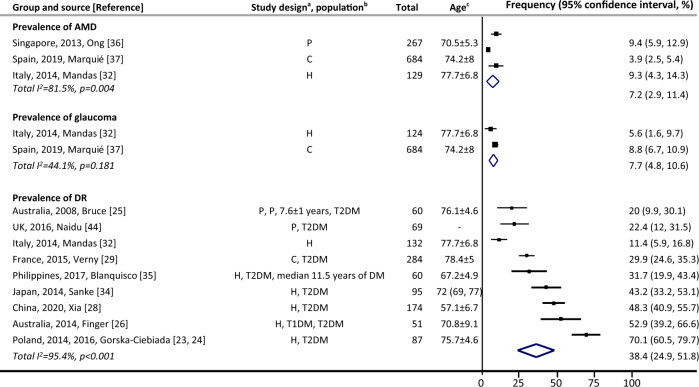
Eye diseases among people with cognitive impairment. AMD denotes age-related macular degeneration, DM diabetes mellitus, DR diabetic retinopathy, T1DM type 1 diabetes mellitus, T2DM type 2 diabetes mellitus, UK United Kingdom. ^a^Case selection: C community-based, H hospital-based, P population-based. All studies did prospective recruitment and cross-sectional data collection. ^b^Population: noted for the prevalence of DR, i.e., whether it was people with T1DM and/or T2DM, or general population if without notes. ^c^Numbers are in years, and listed as mean ± standard, or median (lower and upper interquartile). Point estimates of prevalence reported in the constituent studies are presented using squares. The area of each square is proportional to the study’s weight within each group. Population-based studies were sorted first. The prevalence values are sorted from lowest to highest, with 95% confidence intervals represented by horizontal lines for individual studies and by diamonds for pooled estimates. More details on each individual study can be found in eTable 2.

There were no studies reporting on the incidence. Prevalence of AMD ranged from 3.9% [37] to 9.4% [36] (three studies, Fig. 1). There was no evidence of heterogeneity or high inconsistency between the two studies on prevalence of glaucoma (*p* = 0.18, I^2^ = 44.1%) [32, 37], and the pooled prevalence was 7.7% (95% CI 4.8% to 10.6%). Among people with diabetes and cognitive impairment, prevalence of DR ranged from 20% [25] to 70.1% [23, 24] (eight studies/nine reports). The lowest prevalence of DR (11.4%) was reported in a study where diabetes was not an inclusion criterion [32].

#### Among people with dementia

**Fig. 2:**
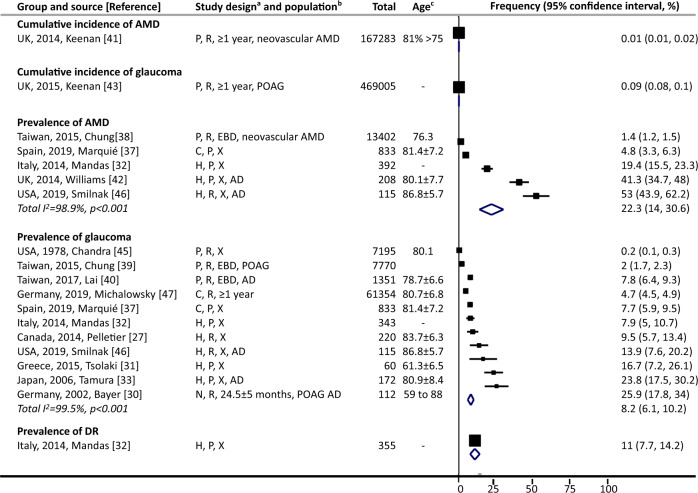
Eye diseases among people with dementia. AD denotes Alzheimer’s disease, AMD age-related macular degeneration, DR diabetic retinopathy, POAG primary open-angle glaucoma, T2DM type 2 diabetes mellitus, UK United Kingdom, USA United States of America. ^a^Study design (three components): (1) case selection: C community-based, H hospital-based, P population-based; (2) recruitment: P prospective, R retrospective; (3) data collection: X represents cross-sectional, otherwise length of follow-up is noted. Numbers are mean ± standard deviation. “EBD” denotes the corresponding eye disease was diagnosed before the diagnosis of dementia. ^b^Population: noted when only specific types of eye disease and/or dementia were investigated in the study; noted for the prevalence of DR, i.e., whether it was people with T2DM, or general population if without notes. ^c^Numbers are in years, and listed as mean, mean ± standard, range, or as specified. Point estimates of prevalence reported in the constituent studies are presented using squares. The area of each square is proportional to the study’s weight within each group. Population-based studies were sorted first. The frequency values are sorted from lowest to highest, with 95% confidence intervals represented by horizontal lines for individual studies and by diamonds for pooled estimates. More details on each individual study can be found in eTable 2.

During follow-ups of “1 year or more”, cumulative incidence of AMD and glaucoma was 0.01% [41] and 0.09% [43], respectively (Fig. 2). Prevalence of AMD and glaucoma ranged from 1.4% [38] to 53% [46] (five studies), and from 0.2% [45] to 25.9% [30] (11 studies), respectively. One study, in which diabetes was not an inclusion criterion, reported prevalence of DR (11%) [32].

### Cognitive impairment or dementia among people with eye diseases

Studies were conducted in Australia [48], Canada [49], China [50], Denmark [51, 52], Japan [53], Malaysia [54], the Netherlands [55], Norway [56], Singapore [57, 58], South Korea [59–61], Taiwan [62–69], Turkey [70–72], the UK [41, 43, 73] and the USA [74–79], and published between 1999 and 2020 (eTable 3). Twenty-eight studies (82.4%) were published in the most recent decade since 2011 [41, 43, 49, 50, 52–54, 57–61, 63–73, 75–79]. There were 21 population-based [41, 43, 48, 51, 55, 57, 58, 60–69, 74, 76–78], one community-based [73], and 12 hospital-based studies [49, 50, 52–54, 56, 59, 70–72, 75, 79]. Other than the frequency, a single study reported on risk factors in univariate analyses, in which higher levels of HbA1c and neuron-specific enolase, and a lower education level increased the risk of cognitive impairment among people with DR [50].

#### Among people with AMD

**Fig. 3:**
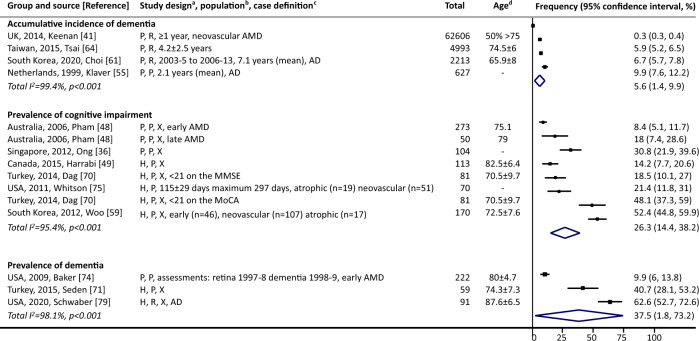
Cognitive impairment or dementia among people with AMD. AD denotes Alzheimer’s disease, AMD age-related macular degeneration, MoCA Montreal Cognitive Assessment, MMSE Mini-Mental State Examination, UK United Kingdom, USA United States of America. ^a^Study design (three components): (1) case selection: C community-based, H hospital-based, P population-based; (2) recruitment: P prospective, R retrospective; (3) data collection: X represents cross-sectional, otherwise length of follow-up is noted. Numbers are mean ± standard deviation. ^b^Population: noted when only a specific type of AMD and/or dementia was investigated in the study. ^c^Case definition: Turkey, 2014, Dag defined cognitive impairment as <21 on the MMSE and MoCA, respectively, and reported the prevalence rates separately. ^d^Numbers are in years, and listed as mean, mean ± standard, or as specified. Point estimates of prevalence reported in the constituent studies are presented using squares. The area of each square is proportional to the study’s weight within each group. Population-based studies were sorted first. The frequency values are sorted from lowest to highest, with 95% confidence intervals represented by horizontal lines for individual studies and by diamonds for pooled estimates. More details on each individual study can be found in eTable 3.

Cumulative incidence of dementia ranged from 0.3% [41] to 9.9% [55] during various follow-up periods (Fig. 3, four studies). Prevalence of cognitive impairment and dementia ranged from 8.4% [48] to 52.4% [59] (six studies) and from 9.9% [74] to 62.6% [79] (three studies), respectively.

#### Among people with glaucoma

**Fig. 4:**
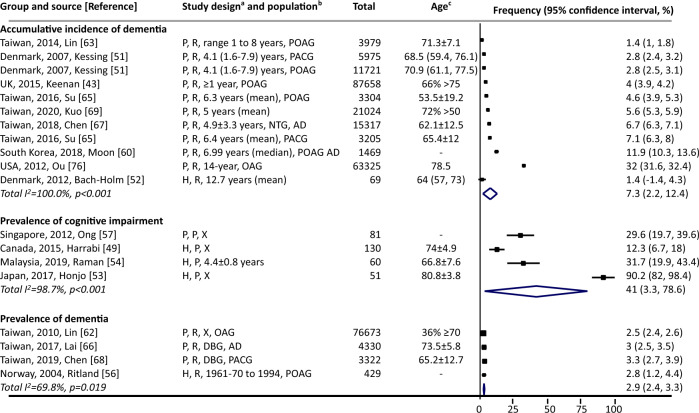
Cognitive impairment or dementia among people with glaucoma. AD denotes Alzheimer’s disease, PACG primary angle-closure glaucoma, POAG primary open-angle glaucoma, UK United Kingdom, USA United States of America. ^a^Study design (three components): (1) case selection: H hospital-based, P population-based; (2) recruitment: P prospective, R retrospective; (3) data collection: X represents cross-sectional, otherwise length of follow-up is noted. Numbers are mean ± standard deviation. ^b^Population: noted when only specific types of glaucoma and/or dementia were investigated in the study. ^c^Numbers are in years, and listed as mean, mean ± standard, median (lower and upper interquartile), or as specified. Point estimates of prevalence reported in the constituent studies are presented using squares. The area of each square is proportional to the study’s weight within each group. Population-based studies were sorted first. The frequency values are sorted from lowest to highest, with 95% confidence intervals represented by horizontal lines for individual studies and by diamonds for pooled estimates. More details on each individual study can be found in eTable 3.

Incidence of dementia was reported in three studies: 2.85 per 1000 person-years among people with POAG [63], and 9.22 [65] and 11.63 [69] per 1000 person-years among people with POAG or PACG. Cumulative incidence of dementia ranged from 1.4% [63] to 32% [76] during various follow-up periods (Fig. 4, 10 studies). Prevalence of cognitive impairment and dementia ranged from 12.3% [49] to 90.2% [53] (four studies) and from 2.5% [62] to 3.3% [68] (four studies), respectively.

#### Among people with DR

**Fig. 5:**
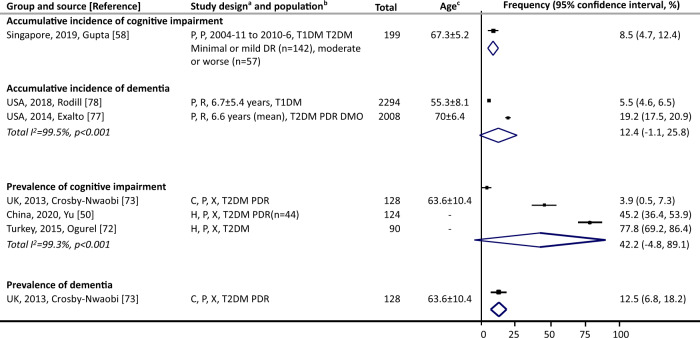
Cognitive impairment or dementia among people with DR. DMO denotes diabetic macular oedema, DR diabetic retinopathy, PDR proliferative diabetic retinopathy, T1DM type 1 diabetes mellitus, T2DM type 2 diabetes mellitus, UK United Kingdom, USA United States of America. ^a^Study design (three components): (1) case selection: C community-based, H hospital-based, P population-based; (2) recruitment: P prospective, R retrospective; (3) data collection: X represents cross-sectional, otherwise length of follow-up is noted. Numbers are mean ± standard deviation. ^b^Population: type of diabetes was noted, also noted when only a specific type of DR was investigated in the study. ^c^Numbers are in years and listed as mean ± standard. Point estimates of prevalence reported in the constituent studies are presented using squares. The area of each square is proportional to the study’s weight within each group. Population-based studies were sorted first. The frequency values are sorted from lowest to highest, with 95% confidence intervals represented by horizontal lines for individual studies and by diamonds for pooled estimates. More details on each individual study can be found in eTable 3.

Cumulative incidence of cognitive impairment was 8.5% (Fig. 5) [58]. Incidence rate of dementia was reported in one study, 32.87 per 1000 person-years [77]. Cumulative incidence of dementia was 5.5% [78] and 19.2% [77] within mean follow-ups of up to 7 years [77, 78]. There were 3.9% [73] to 77.8% [72] of people with DR having cognitive impairment (three studies), and 12.5% [73] of individuals with DR had dementia.

### Associations between eye diseases and cognitive impairment or dementia

One study reported that people with dementia were more likely to have prior neovascular AMD than controls, adjusted OR 1.37 (95% CI 1.14 to 1.65) [38]. Conversely, the associations between AMD, glaucoma, or DR, and cognitive impairment or dementia have been reported in multivariable adjusted models in nine, 10, and three studies, respectively (eFigs. 2–4). In three, eight, and two longitudinal studies, pooled RRs/HRs were 1.46 (95% CI 1.31, 1.61), 1.20 (1.02 to 1.42) and 1.29 (95% CI 1.15, 1.45) for AMD, glaucoma, and DR, respectively.

### Risk of bias

In 48 out of the 56 studies (86%), the target population was considered not representative of the national population with AMD, glaucoma, DR, cognitive impairment or dementia, due to the use of specific inclusion and exclusion criteria (eTable 4a–n), e.g. exclusion of people with a history of cerebrovascular events [27, 64, 72, 73], hearing impairment [26, 28, 75], or not living independently [26, 29]. Among the 16 studies reporting incidence [41, 43, 51, 52, 55, 58, 60, 61, 63–65, 67, 69, 76–78], only four reported all three of the relevant components, i.e., number of incident cases, person-years of follow-up, and incidence rate [63, 65, 69, 77]. Only 21 (22 reports) out of 40 studies reporting on the prevalence reported on all three of the relevant components, i.e., numerator, denominator and prevalence rate [23, 24, 26–28, 30, 31, 33, 34, 37–40, 48, 57, 62, 66, 70–73, 79].

## Discussion

Our work comprehensively reviewed the frequency of coexistent eye disease and cognitive impairment or dementia. The included studies were conducted in 21 countries or regions in Asia (23 studies), Australia (3 studies), Europe (19 studies/20 reports), and North America (11 studies). Most of the studies were conducted in high income countries, with only 13% in upper (6 studies) or lower middle (1 study) income countries. We found heterogeneity across studies with varied frequencies, which was likely due to case mix, e.g., country and ethnicity, various diagnostic criteria or definitions for eye disease and cognitive impairment or dementia, and cross-sectional versus longitudinal study design with various length of follow-up.

### Potential mechanisms influencing the coexistence of eye disease and cognitive impairment

There are several potential mechanisms which have been put forward to explain relationships between eye disease and cognitive function. Whilst a full review of these mechanisms is beyond our systematic summary of the reported levels of co-existence, within the field, we highlight some of the most prominent theories. One such theory is the “use it or lose it” hypothesis in cognitive aging, i.e., a lack of, or reduction in, cognitive, physical, and social activities due to visual impairment has negative consequences for future cognitive function.

Alongside this there may also be common mechanisms operating in eye and cognitive outcomes, e.g., accumulation of similar molecules (for example amyloid β) and pathophysiology, related to both retinal changes and endothelial dysfunction in retinal and cerebral micro-vessels. Besides amyloid β, other molecules that have been found in both drusen and AD plaques include tau protein, proteoglycan, and complement component 3, etc [14]. Chronic inflammation including complement activation, oxidative stress, increased vascular endothelial growth factor in the retinal pigment epithelium and in the cerebrospinal fluid, and decreased clearance of amyloid β have also been suggested as common drivers for AMD and AD [14]. Further, similar retinal changes have been observed in glaucoma and dementia, e.g., decreased retinal thickness, protein deposits, microglial activation, neurodegeneration, and apoptosis [19, 30]. Potential mechanisms also link diabetes and cognitive impairment including a role of the insulin-degrading enzyme (IDE), degrading amyloid β such that amyloid β aggregates in patients with diabetes and hyperinsulinemia as result of lower availability of the IDE [21]. Additionally, in the retina of people with diabetes, micro-vessels are impaired through endothelial dysfunction caused by glycation, oxidative stress, and increased polyol pathway activity, while endothelial dysfunction in the brain affects the development and progression of cerebral small vessel disease associated with the risk of cognitive impairment [21].

### Why knowledge of the co-existence is important

In the current systematic review, population-based data on prevalence were sparse, and estimations were close to, if not lower than, those reported in general populations [2, 4, 11–13]. Thus our summary of the current data does not support population-based based screening of eye diseases in people with cognitive impairment or dementia, or screening of cognitive impairment or dementia in people with AMD, glaucoma, or DR. Indeed, we acknowledge the variations in prevalence estimates for each disease in general populations due to disparities in diagnostic criteria, information sources, and assessment years, etc. For instance, around 11%, 3.2%, and 1.7% Australians aged 55 years and over were affected by AMD, glaucoma, DR, respectively [80], and 8.3% of Australians aged 65 and over had dementia [81], which are slightly different to other estimations [2, 4, 11–13]. Thus, comparisons to rates in general populations should be interpreted with caution.

We did observe that co-existence tended to be more frequent in hospital- or nursing-home based settings potentially because hospital- or nursing-home based populations may be those with more co-morbidities and severe disease, or those who are more likely to seek medical advice from specialists [82]. It is important that clinicians remain alert to the potential need for an eye or cognitive assessment for their older patients. Furthermore, individuals with an eye disease, cognitive impairment, or dementia may be disadvantaged, potentially due to the challenges faced by people who rely on carers and additional help to schedule and attend optometrist appointments (for example, individuals with diabetes who had dementia were 15% less likely to receive an annual eye examination than those without dementia [83]). Therefore, preventative eye checks (or cognitive screening) might be recommended when people with cognitive impairment or dementia (or people with an eye disease) attend their check-ups.

### Measures of the association

Two recent systematic reviews and meta-analyses examined whether AMD, glaucoma and/or DR increase the risk of cognitive decline and/or dementia, and vice versa [84, 85], but the presented evidence is both sparse and mixed. A meta-analysis in 2021 found no evidence for the associations between AMD or glaucoma and risk of dementia [84], but an increased risk of dementia associated with DR, HR 1.34 (95% CI 1.11 to 1.61, pooled estimation of four studies with heterogeneity, *p* = 0.002) [84]. Another meta-analysis in 2019 found that individuals with dementia were at risk of AMD, pooled adjusted OR 1.24 (95% CI 1.04 to 1.47) based on four cross-sectional or case-control studies [85]. Conversely, AMD was also associated with increased risk of cognitive impairment, pooled adjusted OR 2.42 (95% CI 1.06 to 5.56) based on two cross-sectional studies [85]. However, when combining two longitudinal cohort studies with heterogeneity (*p* = 0.01), the association for AMD as a risk factor for AD did not exist, pooled adjusted risk ratio 1.27 (95% CI 0.53 to 3.04) [85]. The current study added to the literature regarding the associations between eye diseases and cognitive impairment or dementia. Longitudinal research is still needed to clarify the causative relationships between eye and cognitive conditions.

### Limitations

Firstly, the validity of the AMD, glaucoma, and DR diagnosis in patients with cognitive impairment or dementia may be questionable, as ocular structural and functional results may be confounded by the presence of comorbidity, such as the likely affected patient cooperation during imaging or visual field testing. Conversely, AMD, glaucoma and DR driven visual impairments may also interfere with cognitive tests, especially when studies adopted cognitive assessment scales containing vision-dependent items to define cognitive impairment. Validated adapted versions of scales, e.g., MMSE [86] and MoCA [87], could be advocated when participants or part of them were visually impaired. However, out of the nine included studies defining cognitive impairment using cut-offs on MMSE or MoCA, an adapted version had been used in only a couple [48, 49].

Secondly, the current data is limited with less than half of the studies being population-based, and only half being longitudinal. Although many studies reported on incident cases did have cumulative incidence data available, incidence rates were available in only four studies. Further, evidence on the risk factors of the comorbidity is sparse, and thus we cannot comment on among those with one condition who are vulnerable to have another. Finally, based on the “use it or lose it” theory and biological associations between eye diseases and cognition, it can be assumed that co-existence would be more pronounced among individuals with severe disease compared to those with mild disease. Also, such coexistence is likely to increase with age, because of the increased prevalence of AMD, POAG, DR, and cognitive impairment or dementia with ageing [2, 4, 11–13]. However, current data are insufficient to explore these further.

### Conclusions and implications

Our review of the co-existence of eye disease and cognitive impairment or dementia provides an important in-depth addition to the evidence base on the frequencies and confirmed the concurrence. We advocate the need for awareness of the comorbid conditions, which deserves appropriate collaborative care across disciplines. Further studies are warranted to collect data on the population-based prevalence and incidence of coexistent eye disease and cognitive impairment or dementia. Our study supports consideration of regular eye examinations or cognitive screening in clinical practice guidelines among older adults with one of these conditions.

## Summary

### What is known about this topic

- There may be relationships between age-related macular degeneration (AMD), glaucoma or diabetic retinopathy (DR) and cognitive impairment or dementia.

### What this study adds

- 56 studies were synthesized.
- Frequencies of co-morbid AMD, glaucoma or DR and cognitive impairment or dementia were quantified.
- More research is required to support or oppose regular eye examinations and cognitive screening among older adults with one of these conditions.

### Supplementary information

Supplementary

## Data Availability

The datasets generated during and/or analysed during the current study are available from the corresponding author on reasonable request.
