# Supplementary material for: Frequency of coexistent eye diseases and cognitive impairment or dementia: a systematic review and meta-analysis
Source: Eye (Lond). 2023 Mar 15;37(15):3128–36. doi: 10.1038/s41433-023-02481-4 (PMC10564749; doi:10.1038/s41433-023-02481-4)
Supplement: Supplementary file 1 — Supplementary [file 41433_2023_2481_MOESM1_ESM.docx]

**Supplementary**

This supplementary document has been provided by the authors to give readers additional information about their work.

Supplement to: Xu Y, Phu J, Aung HL, et al. Frequency of coexistent eye diseases and cognitive impairment or dementia: a systematic review and meta-analysis.

# Appendix A Quality assessment tool

| Quality assessment items | Risk of bias levels | Reasons |
| --- | --- | --- |
| Was the study’s target population a close representation of the national population with age-related macular degeneration, glaucoma, diabetic retinopathy, cognitive impairment, or dementia in relation to relevant variables, e.g. age, sex?  We mainly judged this based on studies` inclusion and exclusion criteria, and based on whether there was anything making the sample unrepresentative of the national population with age-related macular degeneration, glaucoma, diabetic retinopathy, cognitive impairment, or dementia. For incidence or prevalence of age-related macular degeneration, glaucoma, diabetic retinopathy among people with cognitive impairment and/or dementia, target population are people with cognitive impairment and/or dementia, e.g. when they used restricted criteria on e.g. age, sex and cognition (Mini-Mental State Examination 10 to 26 or clinical dementia rating scale 1 to 2), we would say “High risk”. For prevalence of cognitive impairment and/or dementia among people with age-related macular degeneration, glaucoma or diabetic retinopathy, target population are people with age-related macular degeneration, glaucoma or diabetic retinopathy, e.g. when they said “wet age-related macular degeneration only”, we would say “High risk”. | Yes (LOW RISK): The study’s target population was a close representation of the national population. |  |
|  | No (HIGH RISK): The study’s target population was clearly NOT representative of the national population. |  |
| Was the sampling frame a true or close representation of the target population?  We mainly judged this based on how/where studies recruited the sample, or say the “recruiting sites”, and consider “population-based” or “community-based” to be “Low risk”, and “hospital-based” to be “High risk”. | Yes (LOW RISK): The sampling frame was a true or close representation of the target population. |  |
|  | No (HIGH RISK): The sampling frame was NOT a true or close representation of the target population. |  |
| Was some form of random selection used to select the sample, OR, was a census undertaken?  This is about consecutive, random or convenience sampling. Studies with convenience sampling, e.g. “volunteer sample” or a sub sample from an existing study, would be judged as “High risk”. | Yes (LOW RISK): A census was undertaken, OR, some form of random selection was used to select the sample (e.g. simple random sampling, stratified random sampling, cluster sampling, systematic sampling). |  |
|  | No (HIGH RISK): A census was NOT undertaken, AND some form of random selection was NOT used to select the sample. |  |
| Was the likelihood of non-response bias and/or lost to follow-up minimal?  We assumed insurance or general practice registries can be done via data linkage without consent, and would say “Low risk”. Otherwise, for single or multi-center hospital-based studies, when there is no report of responders vs non-responders, we would put “High risk”. | Yes (LOW RISK): The response rate for the study was ≥75%, OR, an analysis was performed that showed no significant difference in relevant demographic characteristics between responders and non-responders. |  |
|  | No (HIGH RISK): The response rate was <75%, and if any analysis comparing responders and non-responders was done, it showed a significant difference in relevant demographic characteristics between responders and non-responders. |  |
| Were data collected directly from the subjects or medical records (as opposed to a proxy)? | Yes (LOW RISK): All data were collected directly from the subjects or their proxy. |  |
|  | No (HIGH RISK): In some instances, data were collected from other investigations (e.g. MRI, EEG). |  |
| Was an acceptable case definition used in the study?  This is about the diagnostic criteria for age-related macular degeneration, diabetic retinopathy, glaucoma, cognitive impairment and/or dementia. For example, for a study investigating prevalence of age-related macular degeneration among people with cognitive impairment, we would check if the diagnostic criteria for age-related macular degeneration and cognitive impairment are both acceptable, and say “High risk” if any of them are unacceptable, e.g. adopting a cut-off on a test score to define mild cognitive impairment, rather than using a standard diagnostic criteria. | Yes (LOW RISK): An acceptable case definition was used. |  |
|  | No (HIGH RISK): An acceptable case definition was NOT used. |  |
| Was the study instrument that measured the parameter of interest (i.e. age-related macular degeneration, glaucoma, diabetic retinopathy, mild cognitive impairment and dementia) shown to have reliability and validity?  We would say “Low risk” only if the diagnosis of age-related macular degeneration, diabetic retinopathy or glaucoma (for studies on incidence or prevalence of age-related macular degeneration, diabetic retinopathy or glaucoma among people with cognitive impairment and/or dementia) and the diagnosis of cognitive impairment and/or dementia (for studies on prevalence of cognitive impairment and/or dementia among people with age-related macular degeneration, diabetic retinopathy or glaucoma) were centrally adjudicated, or say reviewed by the researchers, or positive predictive value etc. have been reported. In addition, incident studies may have included prevalent cases, when people with a relevant disease (i.e. age-related macular degeneration, glaucoma, diabetic retinopathy, cognitive impairment or dementia) were not excluded at baseline or earlier during the follow-up. These studies would also be judged as “High risk”. | Yes (LOW RISK): The study instrument had been shown to have reliability and validity, e.g. centrally adjudicated. |  |
|  | No (HIGH RISK): The study instrument had NOT been shown to have reliability and validity. |  |
| Was the same mode of data collection used for all subjects?  For example, if a study used both prospective and retrospective data collection, we would say “High risk”. | Yes (LOW RISK): The same mode of data collection was used for all subjects. |  |
|  | No (HIGH RISK): The same mode of data collection was NOT used for all subjects. |  |
| Were the numerator(s) and denominator(s) for the parameter of interest appropriate?  For prevalence studies, if all of 1) numerator 2) denominator and 3) prevalence rate were reported without error, we would say “Low risk”. For incidence studies, if all of 1) number of incident cases 2) person-years of follow-up and 3) incidence rate were reported without error, we would say “Low risk”. If we did any calculations to get the number or noticed any errors in reporting, then we would say “High risk”. | Yes (LOW RISK): The paper presented appropriate numerator(s) AND denominator(s) for the parameter of interest (e.g. the prevalence of epileptic seizures or AD). |  |
|  | No (HIGH RISK): The paper did present numerator(s) AND denominator(s) for the parameter of interest but one or more of these were inappropriate. |  |

# Appendix B List of excluded reports with reasons (n = 29)

**Co-existence in context of another disease (n = 3)**

1. Clemons TE, Rankin MW, McBee WL, Age-Related Eye Disease Study Research Group. Cognitive impairment in the Age-Related Eye Disease Study: AREDS report no. 16. Arch Ophthalmol. 2006 Apr;124(4):537-43. doi: 10.1001/archopht.124.4.537.
2. Cumurcu T, Dorak F, Cumurcu BE, Erbay LG, Ozsoy E. Is there any relation between pseudoexfoliation syndrome and Alzheimer’s type dementia? Semin Ophthalmol. 2013 Jul;28(4):224-9. doi: 10.3109/08820538.2013.793726.
3. Liao JL, Xiong ZY, Yang ZK, Hao L, Liu GL, Ren YP, Wang Q, Duan LP, Zheng ZX, Dong J. An association of cognitive impairment with diabetes and retinopathy in end stage renal disease patients under peritoneal dialysis. PLoS One. 2017 Aug 31;12(8):e0183965. doi: 10.1371/journal.pone.0183965.

**Fewer than 50 participants (n = 8)**

1. Bayer AU, Ferrari F. Severe progression of glaucomatous optic neuropathy in patients with Alzheimer’s disease. Eye (Lond). 2002 Mar;16(2):209-12. doi: 10.1038/sj.eye.6700034.
2. Bruce DG, Davis WA, Starkstein SE, Davis TM. Mid-life predictors of cognitive impairment and dementia in type 2 diabetes mellitus: the Fremantle Diabetes Study. J Alzheimers Dis. 2014;42 Suppl 3:S63-70. doi: 10.3233/JAD-132654.
3. Daveckaite A, Grusauskiene E, Petrikonis K, Vaitkus A, Siaudvytyte L, Januleviciene I. Cognitive functions and normal tension glaucoma. Indian J Ophthalmol. 2017 Oct;65(10):974-978. doi: 10.4103/ijo.IJO_756_16.
4. Helmer C, Malet F, Rougier MB, Schweitzer C, Colin J, Delyfer MN, Korobelnik JF, Barberger-Gateau P, Dartigues JF, Delcourt C. Is there a link between open-angle glaucoma and dementia? The Three-City-Alienor cohort. Ann Neurol. 2013 Aug;74(2):171-9. doi: 10.1002/ana.23926.
5. Nolan JM, Loskutova E, Howard AN, Moran R, Mulcahy R, Stack J, Bolger M, Dennison J, Akuffo KO, Owens N, Thurnham DI, Beatty S. Macular pigment, visual function, and macular disease among subjects with Alzheimer’s disease: an exploratory study. J Alzheimers Dis. 2014;42(4):1191-202. doi: 10.3233/JAD-140507.
6. Pankow LJ, Pryor JL, Luchins DJ. Glaucoma screening of patients with and without dementia. J Evid Based Soc Work. 2009 Jan;6(1):29-39. doi: 10.1080/15433710802633338.
7. Roca-Santiago HM, Lago-Bouza JR, Millán-Calenti JC, Gómez-Ulla-Irazazábal F Alzheimer’s disease and age-related macular degeneration [Spanish]. Archivos de la Sociedad Espanola de Oftalmologia, 2006;81(2):73-8.
8. Whitson HE, Ansah D, Whitaker D, Potter G, Cousins SW, MacDonald H, Pieper CF, Landerman L, Steffens DC, Cohen HJ. Prevalence and patterns of comorbid cognitive impairment in low vision rehabilitation for macular disease. Arch Gerontol Geriatr. 2010 Mar-Apr;50(2):209-12. doi: 10.1016/j.archger.2009.03.010.

**Frequency not reported (n = 5)**

1. Ikram MA, Brusselle GGO, Murad SD, van Duijn CM, Franco OH, Goedegebure A, Klaver CCW, Nijsten TEC, Peeters RP, Stricker BH, Tiemeier H, Uitterlinden AG, Vernooij MW, Hofman A. The Rotterdam Study: 2018 update on objectives, design and main results. Eur J Epidemiol. 2017 Sep;32(9):807-850. doi: 10.1007/s10654-017-0321-4.
2. Jefferis JM, Taylor JP, Collerton J, Jagger C, Kingston A, Davies K, Kirkwood T, Clarke MP. The association between diagnosed glaucoma and cataract and cognitive performance in very old people: cross-sectional findings from the Newcastle 85+ Study. Ophthalmic Epidemiol. 2013 Apr;20(2):82-8. doi: 10.3109/09286586.2012.757626.
3. Kurna SA, Akar G, Altun A, Agirman Y, Gozke E, Sengor T. Confocal scanning laser tomography of the optic nerve head on the patients with Alzheimer’s disease compared to glaucoma and control. Int Ophthalmol. 2014 Dec;34(6):1203-11. doi: 10.1007/s10792-014-0004-z.
4. Lee CS, Larson EB, Gibbons LE, Latimer CS, Rose SE, Hellstern LL, Keene CD, Crane PK. Adult Changes in Thought (ACT) Study. Ophthalmology-based neuropathology risk factors: diabetic retinopathy is associated with deep microinfarcts in a community-based autopsy study. J Alzheimers Dis. 2019;68(2):647-655. doi: 10.3233/JAD-181087.
5. Zetterberg M, Landgren S, Andersson ME, Palmér MS, Gustafson DR, Skoog I, Minthon L, Thelle DS, Wallin A, Bogdanovic N, Andreasen N, Blennow K, Zetterberg H. Association of complement factor H Y402H gene polymorphism with Alzheimer’s disease. Am J Med Genet B Neuropsychiatr Genet. 2008 Sep 5;147B(6):720-6. doi: 10.1002/ajmg.b.30668.

**Review or abstracts only (n = 3)**

1. Crosby-Nwaobi R, Sivaprasad S, Forbes A 1. The association between diabetic retinopathy and cognitive impairment. Diabet Med 2013;30(SI): 175-6.
2. Helmer C., Malet F, Rougier MB, Schweitzer C, Colin J, Delyfer MN, Korobelnik J, Barberger-Gateau P, Dartigues J, Delcourt C. Is there a link between open-angle glaucoma and dementia? Results from a prospective population-based cohort study. Alzheimers Dement, 2013;9(S1):756-7. doi: 10.1016/j.jalz.2013.05.1533.
3. Jones-Odeh E, Hammond CJ. How strong is the relationship between glaucoma, the retinal nerve fibre layer, and neurodegenerative diseases such as Alzheimer’s disease and multiple sclerosis? Eye (Lond). 2015 Oct;29(10):1270-84. doi: 10.1038/eye.2015.158.

**Study population not meet inclusion criteria, e.g. participants with dementia grouped with other memory disorders, glaucoma-like alterations not a diagnosis or definition of glaucoma (n = 10)**

1. Asefzadeh B, Rett D, Pogoda TK, Selvin G, Cavallerano A. Glaucoma medication adherence in veterans and influence of coexisting chronic disease. J Glaucoma. 2014 Apr-May;23(4):240-5. doi: 10.1097/IJG.0000000000000044.
2. Brismar T, Maurex L, Cooray G, Juntti-Berggren L, Lindström P, Ekberg K, Adner N, Andersson S. Predictors of cognitive impairment in type 1 diabetes. Psychoneuroendocrinology. 2007 Sep-Nov;32(8-10):1041-51. doi: 10.1016/j.psyneuen.2007.08.002.
3. Cesareo M, Martucci A, Ciuffoletti E, Mancino R, Cerulli A, Sorge RP, Martorana A, Sancesario G, Nucci C. Association between Alzheimer’s disease and glaucoma: a study based on Heidelberg retinal tomography and frequency doubling technology perimetry. Front Neurosci. 2015 Dec;18(9):479. doi: 10.3389/fnins.2015.00479.
4. Ekström C, Kilander L. Pseudoexfoliation and Alzheimer’s disease: a population-based 30-year follow-up study. Acta Ophthalmol. 2014 Jun;92(4):355-8. doi: 10.1111/aos.12184.
5. Jonas JB, Wei WB, Zhu LP, Xu L, Wang YX. Cognitive function and ophthalmological diseases: the Beijing Eye Study. Sci Rep. 2018 Mar 19;8(1):4816. doi: 10.1038/s41598-018-23314-5.
6. Li N, Xu YX, Yan X. Clinical analysis of cognitive function and depressive states in patients with age-related macular degeneration. [Chinese]. International Eye Science 2017;17(10):1905-7.
7. McCoskey M, Addis V, Goodyear K, Sankar PS, Ying GS, Yu Y, Salowe R, Cui QN, Miller-Ellis E, Maguire M, O Apos Brien JM. Association between primary open-angle glaucoma and cognitive impairment as measured by the Montreal Cognitive Assessment. Neurodegener Dis. 2018;18(5-6):315-322. doi: 10.1159/000496233.
8. Rozzini L, Riva M, Ghilardi N, Facchinetti P, Forbice E, Semeraro F, Padovani A. Cognitive dysfunction and age-related macular degeneration. Am J Alzheimers Dis Other Demen. 2014 May;29(3):256-62. doi: 10.1177/1533317513517032.
9. Sahoo S, Thevi T, Soe HHK. Association of Well-Being Index and Cognitive Impairment with Primary Open Angle Glaucoma Patients of Malaysia: A Case-Control Study. Malays J Med Sci. 2018 Feb;25(1):96-100. doi: 10.21315/mjms2018.25.1.11.
10. Salber PR, Selecky CE, Soenksen D, Wilson T. Impact of dementia on costs of modifiable comorbid conditions. Am J Manag Care. 2018 Nov 1;24(11):e344-51.

# Appendix C List of included reports (n = 57)

1. Mandas, A., et al., *Cognitive impairment and age-related vision disorders: Their possible relationship and the evaluation of the use of aspirin and statins in a 65 years-and-over Sardinian population.* Front Aging Neurosci, 2014. **6 (OCT) (no pagination)**(309).

2. Klaver, C.C., et al., *Is age-related maculopathy associated with Alzheimer's Disease? The Rotterdam Study.* Am J Epidemiol, 1999. **150**(9): p. 963-8.

3. Williams, M.A., et al., *The prevalence of age-related macular degeneration in Alzheimer's disease.* J Alzheimers Dis, 2014. **42**(3): p. 909-14.

4. Ong, S.-Y., et al., *Myopia and Cognitive Dysfunction: The Singapore Malay Eye Study.* Investigative Opthalmology & Visual Science, 2013. **54**(1): p. 799.

5. Pham, T.Q., et al., *Relation of Age-Related Macular Degeneration and Cognitive Impairment in an Older Population. [References]*. 2006: Gerontology. Vol.52(6), 2006, pp. 353-358.

6. Baker, M.L., et al., *Early age-related macular degeneration, cognitive function, and dementia: the Cardiovascular Health Study.* Arch Ophthalmol, 2009. **127**(5): p. 667-73.

7. Ong, S.Y., et al., *Visual impairment, age-related eye diseases, and cognitive function: The Singapore Malay Eye Study.* Arch Ophthalmol, 2012. **130**(7): p. 895-900.

8. Woo, S.J., et al., *Cognitive impairment in age-related macular degeneration and geographic atrophy.* Ophthalmology, 2012. **119**(10): p. 2094-101.

9. Seden, D., et al., *Is Alzheimer disease related to age-related macular degeneration?* Turkish Journal of Medical Sciences, 2015. **45**(5): p. 1115-21.

10. Marquié, M., et al., *Visual impairment in aging and cognitive decline: experience in a Memory Clinic.* Sci Rep, 2019. **9**(1).

11. Chung, S.D., et al., *Association between neovascular age-related macular degeneration and dementia: a population-based case-control study in Taiwan.* PLoS ONE [Electronic Resource], 2015. **10**(3): p. e0120003.

12. Choi, S., et al., *Association of Age-Related Macular Degeneration on Alzheimer or Parkinson Disease: A Retrospective Cohort Study.* Am J Ophthalmol, 2020. **210**: p. 41-47.

13. Tsai, D.C., et al., *Age-Related Macular Degeneration and Risk of Degenerative Dementia among the Elderly in Taiwan A Population-Based Cohort Study.* Ophthalmology, 2015. **122**(11): p. 2327-2335.e2.

14. Smilnak, G.J., et al., *Comorbidity of age-related macular degeneration with Alzheimer's disease: A histopathologic case-control study.* PLoS ONE [Electronic Resource], 2019. **14**(9): p. e0223199.

15. Schwaber, E.J., et al., *Co-Prevalence of Alzheimer's Disease and Age-Related Macular Degeneration Established by Histopathologic Diagnosis.* Journal of Alzheimer's disease : JAD., 2020. **19**.

16. Harrabi, H., et al., *Age-related eye disease and cognitive function.* Invest Ophthalmol Vis Sci, 2015. **56**(2): p. 1217-21.

17. Dag, E., et al., *Mini mental state exam versus Montreal cognitive assessment in patients with age-related macular degeneration.* Eur Rev Med Pharmacol Sci, 2014. **18**(20): p. 3025-3028.

18. Keenan, T.D.L., R. Goldacre, and M.J. Goldacre, *Associations between age-related macular degeneration, Alzheimer disease, and dementia: record linkage study of hospital admissions.* JAMA Ophthalmology, 2014. **132**(1): p. 63-68.

19. Whitson, H.E., et al., *Comorbid cognitive impairment and functional trajectories in low vision rehabilitation for macular disease.* Aging Clin Exp Res, 2011. **23**(5/6): p. 343-350.

20. Tsolaki, F., et al., *Helicobacter pylori infection, dementia and primary open-angle glaucoma: are they connected?* BMC Ophthalmol, 2015. **15**: p. 24.

21. Raman, P., et al., *The association between visual field reliability indices and cognitive impairment in glaucoma patients.* J Glaucoma, 2019. **28**(8): p. 685-690.

22. Bayer, A.U., F. Ferrari, and C. Erb, *High occurrence rate of glaucoma among patients with Alzheimer's disease.* Eur Neurol, 2002. **47**(3): p. 165-8.

23. Bach-Holm, D., et al., *Normal tension glaucoma and Alzheimer disease: comorbidity?* Acta Opthalmologica, 2012. **90**(7): p. 683-5.

24. Tamura, H., et al., *High frequency of open-angle glaucoma in Japanese patients with Alzheimer's disease.* J Neurol Sci, 2006. **246**(1-2): p. 79-83.

25. Chung, S.D., et al., *Dementia is associated with open-angle glaucoma: a population-based study.* Eye, 2015. **29**(10): p. 1340-6.

26. Lai, S.W., C.L. Lin, and K.F. Liao, *Glaucoma may be a non-memory manifestation of Alzheimer's disease in older people.* Int Psychogeriatr, 2017. **29**(9): p. 1535-1541.

27. Keenan, T.D., R. Goldacre, and M.J. Goldacre, *Associations between primary open angle glaucoma, Alzheimer's disease and vascular dementia: record linkage study.* Br J Ophthalmol, 2015. **99**(4): p. 524-7.

28. Kessing, L.V., et al., *No increased risk of developing Alzheimer disease in patients with glaucoma.* J Glaucoma, 2007. **16**(1): p. 47-51.

29. Lin, H.C., et al., *Comparison of comorbid conditions between open-angle glaucoma patients and a control cohort: A case-control study.* Ophthalmology, 2010. **117**(11): p. 2088-2095.

30. Lin, I.C., et al., *Glaucoma, Alzheimer's disease, and Parkinson's disease: an 8-year population-based follow-up study.* PLoS ONE [Electronic Resource], 2014. **9**(9): p. e108938.

31. Su, C.W., et al., *Association Between Glaucoma and the Risk of Dementia.* Medicine, 2016. **95**(7): p. e2833.

32. Chen, Y.Y., et al., *Association between normal tension glaucoma and the risk of Alzheimer's disease: a nationwide population-based cohort study in Taiwan.* BMJ Open, 2018. **8**(11): p. e022987.

33. Lai, S.W., C.L. Lin, and K.F. Liao, *Glaucoma correlates with increased risk of Parkinson's disease in the elderly: a national-based cohort study in Taiwan.* Curr Med Res Opin, 2017. **33**(8): p. 1511-1516.

34. Chen, H.-Y. and C.-L. Lin, *Comparison of medical comorbidity between patients with primary angle-closure glaucoma and a control cohort: a population-based study from Taiwan.* BMJ Open, 2019. **9**(3): p. e024209.

35. Kuo, F.H., et al., *Impact of the severities of glaucoma on the incidence of subsequent dementia: A population-based cohort study.* Int J Environ Res Public Health, 2020. **17 (7) (no pagination)**(2426).

36. Ou, Y., et al., *Glaucoma, Alzheimer disease and other dementia: A longitudinal analysis.* Ophthalmic Epidemiol, 2012. **19**(5): p. 285-292.

37. Moon, J.Y., et al., *Association between Open-Angle Glaucoma and the Risks of Alzheimer's and Parkinson's Diseases in South Korea: A 10-year Nationwide Cohort Study.* Sci Rep, 2018. **8**(1): p. 11161.

38. Pelletier, A.A., et al., *Prevalence of glaucoma in hospitalized older adults with Alzheimer's disease.* Can J Neurol Sci, 2014. **41**(2): p. 206-9.

39. Michalowsky, B., W. Hoffmann, and K. Kostev, *Association Between Hearing and Vision Impairment and Risk of Dementia: Results of a Case-Control Study Based on Secondary Data.* Front Aging Neurosci, 2019. **11**.

40. Chandra, V., N.E. Bharucha, and B.S. Schoenberg, *Conditions associated with Alzheimer's disease at death: case-control study.* Neurology, 1986. **36**(2): p. 209-11.

41. Honjo, M., et al., *The association between structure-function relationships and cognitive impairment in elderly glaucoma patients.* Sci Rep, 2017. **7**(1): p. 7095.

42. Ritland, J.S., et al., *Exfoliative glaucoma and primary open-angle glaucoma: associations with death causes and comorbidity.* Acta Ophthalmol Scand, 2004. **82**(4): p. 401-4.

43. Bruce, D.G., et al., *Predictors of cognitive impairment and dementia in older people with diabetes.* Diabetologia, 2008. **51**(2): p. 241-248.

44. Naidu, V.V., et al., *Associations between Retinal Markers of Microvascular Disease and Cognitive Impairment in Newly Diagnosed Type 2 Diabetes Mellitus: A Case Control Study.* PLoS One, 2016. **11**(1): p. e0147160.

45. Yu, Z.W., et al., *High serum neuron-specific enolase level is associated with mild cognitive impairment in patients with diabetic retinopathy.* Diabetes, Metabolic Syndrome and Obesity: Targets and Therapy, 2020. **13**: p. 1359-1365.

46. Ogurel, T., et al., *Mini-mental state exam versus Montreal Cognitive Assessment in patients with diabetic retinopathy.* Niger J Clin Pract, 2015. **18**(6): p. 786.

47. Blanquisco, L.R., et al., *Factors associated with mild cognitive impairment among elderly filipinos with type 2 diabetes mellitus.* Journal of the ASEAN Federation of Endocrine Societies, 2017. **32**(2): p. 145-150.

48. Finger, R.P., et al., *Near Vision Impairment Is Associated With Cognitive Impairment in Type 2 Diabetes.* Asia Pac J Ophthalmol (Phila), 2014. **3**(1): p. 17-22.

49. Gupta, P., et al., *Association between diabetic retinopathy and incident cognitive impairment.* Br J Ophthalmol, 2019. **103**(11): p. 1605-1609.

50. Crosby-Nwaobi, R.R., et al., *The relationship between diabetic retinopathy and cognitive impairment.* Diabetes Care, 2013. **36**(10): p. 3177-3186.

51. Exalto, L.G., et al., *Severe diabetic retinal disease and dementia risk in type 2 diabetes.* J Alzheimers Dis, 2014. **42 Suppl 3**: p. S109-17.

52. Rodill, L.G., et al., *Diabetic Retinopathy and Dementia in Type 1 Diabetes.* Alzheimer Dis Assoc Disord, 2018. **32**(2): p. 125-130.

53. Shan Xia, S., et al., *The factors contributing to cognitive dysfunction in type 2 diabetic patients.* Annals of Translational Medicine, 2020. **8**(4).

54. Verny, C., et al., *Prevalence of cognitive decline and associated factors in elderly type 2 diabetic patients at inclusion in the GERODIAB cohort.* Eur Geriatr Med, 2015. **6**(1): p. 36-40.

55. Sanke, H., et al., *Relationship between olfactory dysfunction and cognitive impairment in elderly patients with type 2 diabetes mellitus.* Diabetes Res Clin Pract, 2014. **106**(3): p. 465-473.

56. Gorska-Ciebiada, M., et al., *Adiponectin, leptin and IL-1 beta in elderly diabetic patients with mild cognitive impairment.* Metab Brain Dis, 2016. **31**(2): p. 257-66.

57. Gorska-Ciebiada, M., et al., *Mild cognitive impairment and depressive symptoms in elderly patients with diabetes: prevalence, risk factors, and comorbidity.* Journal of Diabetes Research, 2014. **2014**: p. 179648.

# Appendix D Diagnostic criteria or definitions

**Eye diseases**

Nineteen studies reported on age-related macular degeneration (AMD).^1-19^ AMD assessments were detailed in 15 studies, including using 1) retinal photograph grading according to the International Age-Related Maculopathy (ARM) epidemiological study group,^1-3^ Wisconsin ARM Grading system,^4-7^ Age-Related Eye Disease Study classification system;^8, 9^ or the American Academy of Ophthalmology Age-Macular Degeneration Preferred Practice Patterns guidelines;^10^ 2) International Classification of Diseases (ICD) codes;^11-13^ and 3) Sarks grades to assess retinal specimens.^14, 15^ It was unclear how AMD was defined in another four studies.^16-19^ Two studies focused on neovascular AMD.^11, 18^

Twenty-eight studies reported on glaucoma.^1, 7, 10, 14, 16, 20-42^ Glaucoma asssessments were based on at least one of: intraocular pressure,^7, 10, 20-24^ characteristics of the anterior chamber^7, 24^ and/or the optic nerve head,^7, 10, 22, 24^ visual field tests,^7, 10, 20-23^ or histopathology^14^ in eight studies. Specific criteria in these studies included criteria adopted in the Thessaloniki Eye Study,^20^ Hodapp-Parrish-Anderson Glaucoma Grading Scale,^21^ and International Society Geographical and Epidemiological Ophthalmology scheme.^7^ Glaucoma was coded using ICD codes in 12 studies,^25-36^ and the Korean Classification of Diseases code in one study.^37^ It was unclear how glaucoma was defined in seven studies.^1, 16, 38-42^ Six studies focused on POAG.^22, 25, 27, 29, 30, 34, 37, 42^ Two studies reported the frequency of dementia among POAG and PACG populations separately.^28, 31^

Fifteen studies (16 reports) reported on diabetic retinopathy (DR).^1, 43-57^ Study ophthalmologists made the DR diagnosis in five studies, based on Early Treatment of Diabetic Retinopathy Study grading criteria^46, 47^ and/or International Clinical Diabetic Retinopathy Disease Severity Scales,^47^ or any retinal haemorrhage or microaneurysm found in either eye.^43-45^ In three studies, DR was defined based on grading of the retinal photograph according to the modified Airlie House Classification System.^48-50^ ICD codes were used in two studies.^51, 52^  Assessment details were not provided in five studies (six reports).^1, 53-57^ Frequencies of cognitive impairment and dementia were only reported in people with proliferative DR in one study,^50^ and another study only included proliferative DR and diabetic macular edema.^51^

**Cognitive impairment**

Cognitive impairment was assessed or defined in 22 studies (23 reports), using 1) diagnostic criteria for mild cognitive impairment (MCI, used in six studies (seven reports));^1, 8, 10, 45, 53, 56, 57^ and 2) cut-offs on varied cognitive testing scales (16 studies).^4, 5, 7, 16, 17, 19, 21, 41, 44, 46-50, 54, 55^ Diagnostic criteria included the National Institute on Aging-Alzheimer’s Association (NIA-AA) and/or Petersen’s criteria,^1, 10, 45, 53, 56, 57^ and the International Working Group on MCI.^8^ The cognitive assessment scales included the Mini-Mental State Examination (MMSE) or Montreal Cognitive Assessment (MoCA),^5, 16, 17, 41, 46, 47, 50, 54, 55^ Abbreviated Mental Test,^4, 7, 49^ Modified Telephone Interview for Cognitive Status,^19, 44^ Clock Drawing Test,^21^ and Short Orientation-Memory-Concentration Test.^48^

Thirty-seven studies reported on Dementia.^1-3, 6, 9-15, 18, 20, 22-40, 42, 43, 50-52^ Dementia was assessed according to: 1) diagnostic criteria (13 studies);^1-3, 6, 9, 10, 14, 15, 20, 22, 24, 33, 38^ 2) ICD codes (16 studies);^11-13, 23, 25-32, 34-36, 40^ and 3) the Korean Classification of Diseases codes.^37^ One study defined dementia as present in those scoring 0 to 2 on the Mini-Cog.^50^ In another six studies, it was unclear how dementia was defined.^18, 39, 42, 43, 51, 52^ Diagnostic criteria included the Diagnostic and Statistical Manual of Mental Disorders,^6, 10, 38^ the National Institute of Neurological and Communicative Disorders and Stroke and the Alzheimer's Disease and Related Disorders Association (NINCDS-ADRDA)`s Alzheimer's Disease (AD) criteria,^1-3, 9, 20, 22, 24^ Braak and Braak system to assess brain specimens,^14, 15^ the NIA-AA diagnostic guidelines,^38^ the Association Internationale pour la Recherche et l’Enseignement en Neurosciences (NINDS-AIREN) criteria for Vascular dementia (VaD),^1^ and relavant criteria for dementia associated with Parkinson’s disease, dementia with Lewy bodies and frontotemporal lobar dementia.^20^ Twelve studies focused on AD.^2, 3, 9, 12, 14, 15, 22, 24, 26, 32, 37, 38^

# eFigure 1 Flow diagram for systematic review

Records identified through database searching
(n = 6,803)

**Screening**

**Identification**

**Included**

**Eligibility**

Records after duplicates removed
(n = 5,496)

Records screened
(n = 5,496)

Records excluded
(n = 5,422)

Full-text reports assessed for eligibility (n = 74)

Full-text reports excluded, with reasons (n = 29)

Co-existence in context of another disease (n = 3)

Fewer than 50 participants (n = 8)

Frequency not reported (n = 5)

Review, abstracts only (n = 3)

Study population not meet inclusion criteria (e.g. participants with dementia grouped with “other memory disorders”, glaucoma-like alterations not a diagnosis or definition of glaucoma) (n = 10)

Reports identified through reference lists or citation trails
(n = 12)

Studies included in synthesis
57 reports (56 studies)


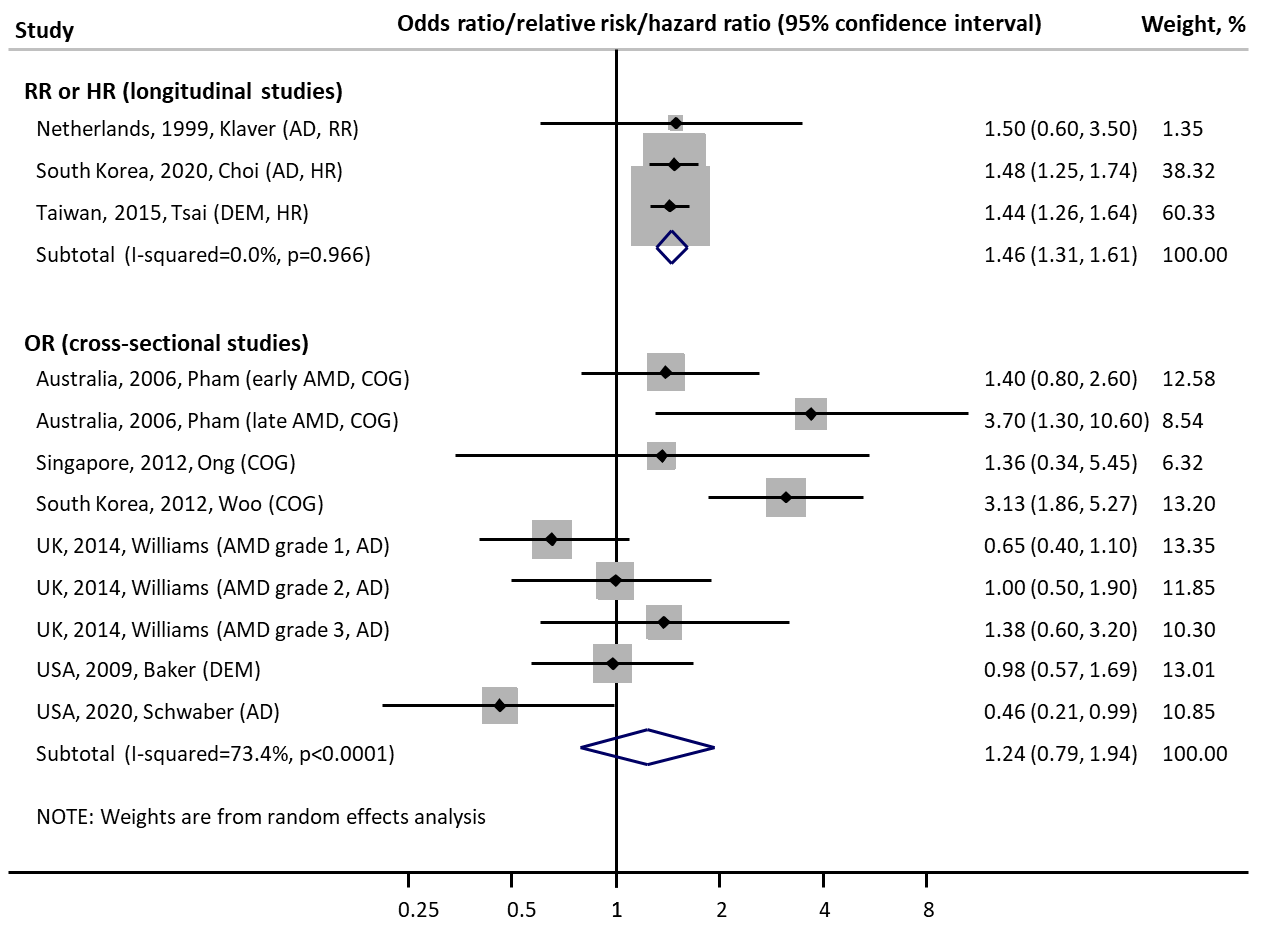


# eFigure 2 Forest plot for the multivariable-adjusted associations between age-related macular degeneration and cognitive impairment or dementia

Meta-analyses were conducted separately by relative risk (RR) or hazard ratio (HR), and odds ratio (OR). In the brackets in the Study column, it noted whether the risk factor, age-related macular degeneration (AMD), was early or late, etc, if specified in the study, whether the outcome was cognitive impairment (COG) or dementia (DEM) including Alzheimer’s disease (AD), and whether the associations were reported as RR or HR.

Adjusted variables were: *Netherlands, 1999, Klaver,* age, sex, smoking, atherosclerosis; *South Korea, 2012, Woo*, age, education, visual acuity; *South Korea, 2020, Choi,* age, sex, household income, smoking, alcohol consumption, physical activity, BMI, SBP, fasting serum glucose, total cholesterol, Charlson comorbidity index; *Taiwan, 2015, Tsai*, Parkinson’s disease, hypertension, diabetes, dysrhythmia, coronary artery diseases, hyperlipidemia, thyroid diseases, number of National Health Insurance claims for outpatient visits; *Australia, 2006, Pham*, age, sex, visual impairment, stroke, current smoking status, hypertension, alcohol consumption, post-high-school qualification; *Singapore, 2012, Ong* age, sex, education level, income category, type of housing, cataract, DR, glaucoma*; South Korea, 2012, Woo* age, education, visual acuity; *UK, 2014, Williams,* age, smoking, APOE genotypes, "generally unwell recently"; *USA, 2009, Baker,* age, sex, ethnicity, study center, education (completed high school), SBP, total cholesterol level, diabetes, smoking, APOE; *USA, 2020, Schwaber*, sex, race, glaucoma, diabetes, hypertension, depression, atherosclerosis.


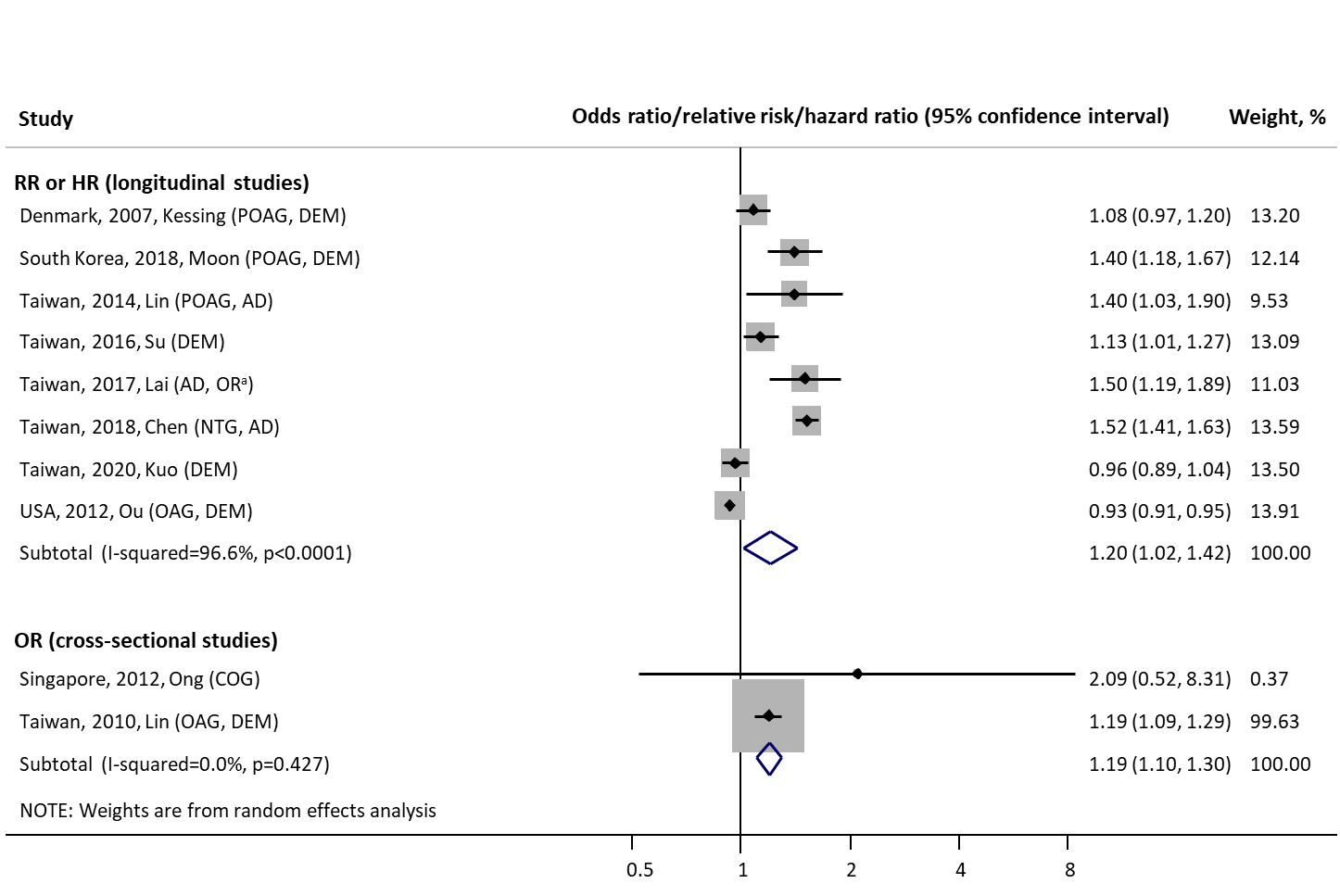


# eFigure 3 Forest plot for the multivariable-adjusted associations between glaucoma and cognitive impairment or dementia

Meta-analyses were conducted separately by relative risk (RR) or hazard ratio (HR), and odds ratio (OR). In the brackets in the Study column, it noted whether the risk factor, glaucoma, was primary open angle glaucoma (POAG), open angle glaucoma (OAG), normal tension glaucoma (NTG), if specified in the study, whether the outcome was cognitive impairment (COG) or dementia (DEM) including Alzheimer’s disease (AD), and whether the associations were reported as relative risk (RR) or hazard ratio (HR).

^a^This longitudinal study (doi: 10.1017/S1041610217000801) reported the OR using logistic regression model and was considered as RR.

Adjusted variables were: *Denmark, 2007, Kessing,* age at index diagnosis, sex, time from discharge, substance use; *South Korea, 2018, Moon,* age, sex, residential area, income, Charlson comorbidity index, hypertension, diabetes mellitus, hyperlipidemia, ischemic stroke; *Taiwan, 2014, Lin,* age, sex, hypertension, diabetes, heart failure, stroke, insurance eligibility group, monthly income, diagnostic year, urbanization level, Charlson comorbidities index; *Taiwan, 2016, Su,* age, sex, hypertension, DM, CAD, hyperlipidemia, head injury; *Taiwan, 2017, Lai,* age*; Taiwan, 2018, Chen,* age, sex, diabetes, hypertension, hyperlipidemia, CAD, stroke; *Taiwan, 2020, Kuo,* age, sex, education, marital status, hypertension, DM, IHD, hyperlipidemia, congestive heart failure, PVD, cerebrovascular disease, AMD, sensorineural hearing loss, hemiplegia or paraplegia; *USA, 2012, Ou* age, sex, race, Charlson comorbidity index, AMD, DR, PDR, vitreous hemorrhage, cataract, pseudophakia/aphakia, cataract surgery; *Singapore, 2012, Ong,* age, sex, education level, income category, type of housing, cataract, DR, glaucoma; *Taiwan, 2010, Lin,* age, sex, monthly income, level of urbanization of the community in which the patient resided.


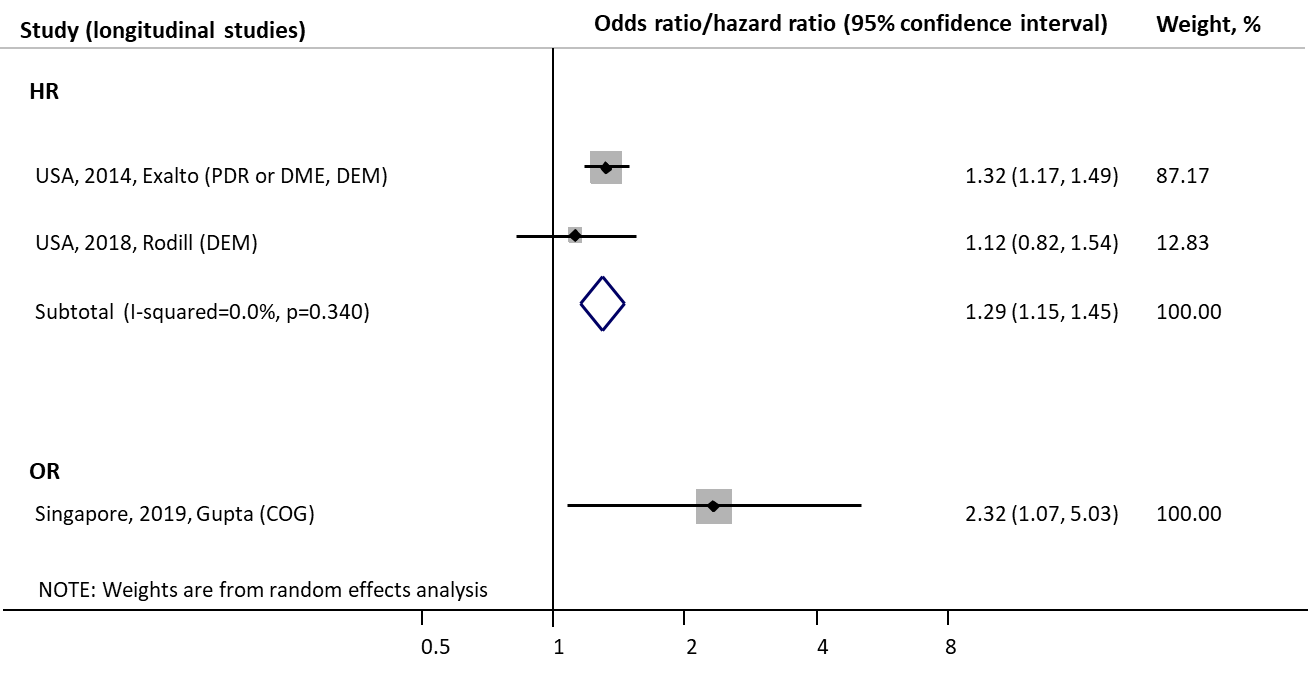


# eFigure 4 Forest plot for the multivariable-adjusted associations between diabetic retinopathy and cognitive impairment or dementia

Meta-analyses were conducted separately by hazard ratio (HR) and odds ratio (OR). In the brackets in the Study column, it noted whether the risk factor, diabetic retinopathy, was Proliferative diabetic retinopathy (PDR) or Diabetic macular edema (DME), if specified in the study, and whether the outcome was cognitive impairment (COG) or dementia (DEM).

Adjusted variables were: *USA, 2014, Exalto*, age, sex, race, education, medication, diabetes duration, HbA1c, insulin, hypoglycemic or hyperglycemia events, cerebrovascular disease, hypertension, hyperlipidemia, PAD, MI, congestive heart failure, BMI, smoking; *USA, 2018, Rodill*, age, sex, race, glycosylated hemoglobin, neuropathy, diabetic nephropathy, end-stage renal disease, CVD, stroke, hyperglycemic or hypoglycemic episodes; *Singapore, 2019, Gupta*, age, sex, race, education, income, spherical equivalent, HbA1c, diabetes duration, hypertension, CVD, cataract, AMD, glaucoma, undercorrected refractive error in the better eye, better eye presenting visual acuity.

# eTable 1 Description of search strategy and results (1 June 2020, n = 6,803)

| **Database** | **Search strategy** | **Number of reports** |
| --- | --- | --- |
| **Medline** | 1. macular degeneration/ or geographic atrophy/ or macular edema/ or vitelliform macular dystrophy/ or wet macular degeneration/ | 23915 |
|  | 2. exp Retinal Degeneration/ | 42104 |
|  | 3. ((macul* or retina* or choroid*) adj4 degener*).tw. | 26485 |
|  | 4. ((macul* or geographic) adj3 dystroph*).tw. | 1495 |
|  | 5. ((macul* or geographic) adj2 atroph*).tw. | 1624 |
|  | 6. (maculopath* or drusen* or retinal neovascularization* or choroidal neovascularization*).tw. | 11968 |
|  | 7. 1 or 2 or 3 or 4 or 5 or 6 | 56831 |
|  | 8. exp Ocular Hypertension/ | 54720 |
|  | 9. (glaucoma or (ocular or eye hyperten*)).tw. | 149123 |
|  | 10. 8 or 9 | 161277 |
|  | 11. Diabetic Retinopathy/ | 24328 |
|  | 12. ((diabetes or diabetic or diabetes mellitus) and (retinopath* or eye disease* or macular disease* or macular edema)).tw. | 27409 |
|  | 13. 11 or 12 | 33750 |
|  | 14. exp dementia/ | 164032 |
|  | 15. cognitive dysfunction/ | 16607 |
|  | 16. (dementia* or Alzheimer*).tw. | 180293 |
|  | 17. 14 or 15 or 16 | 227661 |
|  | 18. 7 or 10 or 13 | 234076 |
|  | 19. 17 and 18 | 1364 |
|  | 20. limit 19 to humans | **1240** |
| **EMBASE** | 1. macular degeneration/ or geographic atrophy/ or macular edema/ or vitelliform macular dystrophy/ or wet macular degeneration/ | 10849 |
|  | 2. exp retina degeneration/ | 43863 |
|  | 3. ((macul* or retina* or choroid*) adj4 degener*).tw. | 42294 |
|  | 4. ((macul* or geographic) adj3 dystroph*).tw. | 2228 |
|  | 5. ((macul* or geographic) adj2 atroph*).tw. | 2899 |
|  | 6. (maculopath* or drusen* or retinal neovascularization* or choroidal neovascularization*).tw. | 18259 |
|  | 7. 1 or 2 or 3 or 4 or 5 or 6 | 80515 |
|  | 8. exp glaucoma/ | 228811 |
|  | 9. (glaucoma or (ocular or eye hyperten*)).tw. | 227028 |
|  | 10. 8 or 9 | 252113 |
|  | 11. exp diabetic retinopathy/ | 45133 |
|  | 12. ((diabetes or diabetic or diabetes mellitus) and (retinopath* or eye disease* or macular disease* or macular edema)).tw. | 47487 |
|  | 13. 11 or 12 | 60725 |
|  | 14. 7 or 10 or 13 | 366104 |
|  | 15. exp cognitive defect/ | 495394 |
|  | 16. (dementia* or Alzheimer*).tw. | 300319 |
|  | 17. 15 or 16 | 537666 |
|  | 18. 14 and 17 | 5454 |
|  | 19. limit 18 to human | **4577** |
| **PsycINFO** | 1. ((macul* or retina* or choroid*) adj4 degener*).tw. | 1315 |
|  | 2. ((macul* or geographic) adj3 dystroph*).tw. | 22 |
|  | 3. ((macul* or geographic) adj2 atroph*).tw. | 19 |
|  | 4. (maculopath* or drusen* or retinal neovascularization* or choroidal neovascularization*).tw. | 99 |
|  | 5. 1 or 2 or 3 or 4 | 1395 |
|  | 6. glaucoma/ | 464 |
|  | 7. (glaucoma or (ocular or eye hyperten*)).tw. | 7873 |
|  | 8. 6 or 7 | 7877 |
|  | 9. ((diabetes or diabetic or diabetes mellitus) and (retinopath* or eye disease* or macular disease* or macular edema)).tw. | 567 |
|  | 10. cognitive impairment/ or exp dementia/ or Alzheimer’s disease/ | 102033 |
|  | 11. (dementia* or Alzheimer*).tw. | 100566 |
|  | 12. 10 or 11 | 123891 |
|  | 13. 5 or 8 or 9 | 9487 |
|  | 14. 12 and 13 | 334 |
|  | 15. Limit 14 to Human | **283** |
| **CINAHL** | 1. MM “Macular Degeneration” | 5,070 |
|  | 2. ‘macul*’ or ‘retina*’ or ‘choroid*’ adj4 ‘degener*’.tw. | 29,442 |
|  | 3. ‘macul*’ or ‘geographic’ adj3 ‘dystroph*’.tw. | 13,490 |
|  | 4. ‘macul*’ or ‘geographic’ adj2 ‘atroph*’.tw. | 13,490 |
|  | 5. ‘maculopath*’ or ‘drusen*’ or ‘retinal neovascularization*’ or ‘choroidal neovascularization*’.tw. | 1,034 |
|  | 6. MH “Ocular Hypertension+” | 8,952 |
|  | 7. ‘glaucoma’ or ‘ocular’ or ‘eye hyperten*’.tw. | 26,630 |
|  | 8. MM “Diabetic Retinopathy” | 4,101 |
|  | 9. ‘diabetes’ or ‘diabetic’ or ‘diabetes mellitus’ and ‘retinopath*’ or ‘eye disease*’ or ‘macular disease*’ or ‘macular edema’.tw | 251,961 |
|  | 10. S1 OR S2 OR S3 OR S4 OR S5 OR S6 OR S7 OR S8 OR S9 | 254,150 |
|  | 11. (MH “Cognition Disorders”) or (MH “Dementia+”) | 100,812 |
|  | 12. ‘dementia*’ or ‘Alzheimer*’ | 95,616 |
|  | 13. S11 OR S12 | 118,853 |
|  | 14. S10 AND S13 | 5,005 |
|  | 15. Exclude MEDLINE records and Limit to Human | **703** |

# eTable 2 Characteristics of studies on the frequency of age-related macular degeneration, glaucoma, or diabetic retinopathy among people with cognitive impairment or dementia

| **Source^a,b,c^** | **Inclusion and exclusion criteria** | **Cognitive impairment or dementia diagnostic criteria, type, and severity** | **AMD, glaucoma or DR diagnostic criteria, type and stage** | **Subgroup, study name, recruitment period, average length of follow-up (mean±SD)** | **Association between cognitive impairment or dementia and AMD, glaucoma, or DR (95%CI)** |
| --- | --- | --- | --- | --- | --- |
| Australia, 2008, Bruce^43P, P^ | People reside in the study catchment area with a clinician-verified diagnosis of T2DM during initial screening, and aged ≥70. Excluded people who died before recruitment, could not be contacted and declined participation. | MMSE score <28/30 or a score ≥3.1 on the Informant Questionnaire for Cognitive Decline in the Elderly (IQCODE), or subjective memory loss. | Retinopathy was defined as any grade of retinopathy detected by direct and/or indirect ophthalmoscopy in one or both eyes and/or more detailed assessment by an ophthalmologist. | Prevalence of DR among cognitive impairment, 1993-1996 (initial screening for DM, and in 2008-2011 to assess for cognitive impairment or dementia), Fremantle Diabetes Study, 7.6±1 years. | 20.3% of those with cognitive impairment versus 13.3% of those with normal cognitive function had DR (non-significant). |
| Australia, 2014, Finger^48H, P, X^ | T1DM or T2DM, aged ≥18, with and without DR, English-speaking, free of significant hearing and cognitive impairment, and living independently. | 6-item cognitive impairment test, also known as the Short Orientation-Memory-Concentration Test, with a cut-off of 8. | modified Airlie House Classification System. Non-proliferative and proliferative DR. | Prevalence of DR among cognitive impairment, 2009-2010, Diabetes Management Project. | Did not find an association of cognitive impairment with DR (p=0.471). |
| Canada, 2014, Pelletier^38H, R, X^ | A diagnosis of AD or mixed dementia either before or during the period of hospitalization. Excluded those with other form of dementia, massive stroke, and long-term use of corticosteroids. | DSM-IV and recommendations from the NIA-AA workgroups on diagnostic guidelines for AD. | - | Prevalence of glaucoma among dementia, 2008-2009. | Glaucoma was significantly more prevalent in the those admitted to hospital with AD or mixed dementia (9.5%) than in the control group (4.1%, p=0.023). |
| China, 2020, Xia^53H, P, X^ | T2DM, aged 45-74, minimal education level of 6 years, symptoms of memory deficits confirmed by others, and visually and auditory fit to take neuropsychological test. Excluded those with any metabolic diseases that may affect cognitive function temporarily, such as acute carbohydrate metabolic events in the last 3 months, including severe hypoglycemia, diabetic ketoacidosis, diabetic hyperglycemia hypertonic coma, hypothyroidism, etc., history of head trauma, cerebral intake, mental and neurological disorders such as depression, anxiety, delirium, severe lung or kidney diseases, heart failure, malignant tumors, etc., history of drug dependence, used any of antidepressant drugs, anti-Parkinson drugs, anti-epileptic drugs, sedative hypnotic drugs, etc. in the last month, using cognitive dysfunction medications such as donepezil, memantine, etc. | Core clinical criteria for MCI from the NIA-AA and Petersen’s criteria: concern in terms of a change in cognitive performance, and impairment in ≥1 cognitive domain, and preservation of independence in social functional abilities, and not demented. | - | Prevalence of DR among cognitive impairment, 2018-2019. | There was no significant difference in DR between people with MCI and those with normal cognition (p=0.68). |
| France, 2015, Verny^54C, P, X^ | Aged ≥70 diabetic patients, activities of daily living score ≥3/6 and no concurrent acute conditions. Excluded those with T1DM or secondary DM, loss of autonomy (activities of daily living score <3/6), acute disease stage (transitory exclusion criterion) and refusal to participate in the study. | Cognitive disorders were defined by previously known dementia and/or MMSE scores ≤24/30. | - | Prevalence of DR among cognitive impairment, GERODIAB study, 2009-2010. | Similar rates of people with (29.9%) and without cognitive impairment (24.5%) had DR, p=0.077. |
| Germany, 2002, Bayer^22N, R^ | Meeting diagnostic criteria of probable AD by the NINCDS-ADRDA. Excluded those with uncontrolled DM, optic nerve head not visible and unreliable perimetry (bilateral cataract), AD associated with Parkinson’s disease. | NINCDS-ADRDA criteria. AD duration ranged from 8 months to 10 years, early onset AD (before the age of 65, n=38) and late onset AD (after 65 years, n=74). | A characteristic pattern of glaucomatous visual field loss or a cup-to-disc ratio of ≥0.8 with an optic nerve head appearance consistent with glaucoma. POAG (including high tension and low tension). | Prevalence of glaucoma among dementia, 24.5±5 months. | 25.9% AD patients had probable glaucoma, significantly higher than the 5.2% in the control group. |
| Germany, 2019, Michalowsky^39C, R^ | Aged ≥65, observation time of ≥12 months prior to the date diagnosed with dementia. | - | - | Prevalence of glaucoma among dementia, 2013-2017, Disease Analyzer database, ≥1 year | No differences in the rates of glaucoma between people with (4.7%) and without (5%) dementia, p=0.11. |
| Greece, 2015, Tsolaki^20H, P, X^ | Patients with dementia (n=60), including official diagnosis of AD (n=36), dementia with Parkinson’s disease (n=9), Lewy body dementia (n=9), frontotemporal dementia (n=6). | NINCDS-ADRDA, clinical diagnostic criteria for dementia associated with Parkinson’s disease, consensus guidelines for the clinical and pathological diagnosis of dementia with Lewy bodies and frontotemporal lobar degeneration: a consensus on clinical diagnostic criteria. | Criteria from the Thessaloniki Eye Study. | Prevalence of glaucoma among dementia. | Frequency of dementia in people with glaucoma 16.7% was higher than that in the control group 0%, p<0.01. |
| Italy, 2014, Mandas^1H, P, X^ | Aged ≥65. | Symptomatic pre-dementia with preserved ability to function independently in daily life, were referred to as MCI (MMSE: mean±SD 24±2.6). Possible/probable diagnosis of AD according to the NINCDS-ADRDA, possible/probable VaD according to the NINDS-AIREN, differential diagnosis between AD, VaD, and mixed dementia was always supported by neuroimaging evidence (magnetic resonance scan and/or nuclear magnetic resonance) (MMSE: 17.0±5.4 (AD), 17.3±4.5 (mixed dementia), 18.0±4.8 (VaD)). | AMD macular degeneration international classification grading system. Glaucoma diagnostic criteria unclear. | Prevalence of AMD, glaucoma, or DR among cognitive impairment or dementia, 2006-2013. | AMD and DR were associated with cognitive impairment or dementia unadjusted OR 1.9 (1.3 to 3.0) and 2.0 (1.1 to 3.4), respectively. Glaucoma was not associated with cognitive impairment or dementia unadjusted OR 1.4 (0.7 to 25). |
| Japan, 2006, Tamura^24 H, P, X^ | Patients with AD institutionalized in 4 Japanese hospitals or visited those hospitals. | NINCDS-ADRDA criteria. | POAG width of the angle of the anterior chamber >grade 2 (method of Van Herick et al.), a vertical cup-to-disc ratio of the optic nerve head >0.7, and/or difference between the vertical cup-to-disc ratio in the eyes >0.2 with characteristic glaucomatous disc change. | Prevalence of glaucoma among dementia. | POAG was found in 41 (23.8%) of the AD patients, which was a significantly (p=0.0002) higher than that in the controls (9.9%). |
| Japan, 2014, Sanke^55H, P, X^ | T2DM, aged ≥65 and free of clinically evident cognitive impairment. Excluded patients with severe infections within the past 2 weeks, scheduled for surgery or who had undergone surgery, with severe trauma, with psychiatric disorders, hypothyroidism, or brain tumors, with partial or complete olfactory dysfunction associated with sinusitis, allergic rhinitis, and deviated nasal septum, on steroid treatment, with an MMSE score of <18. | “Possible cognitive impairment group (24≤MMSE≤26)” and “Probable dementia group (18≤MMSE≤23)” defined in this study were considered as cognitive impairment definition. | - | Prevalence of DR among cognitive impairment, 2012-2013. | No association between DR and cognitive impairment p=0.055. |
| Philippines, 2017, Blanquisco^47H, P, X^ | Aged ≥60, T2DM of any duration, literate, independent in activities of daily living, without known neurocognitive, neurologic or psychiatric disorder, intracranial neoplasm, infectious disease, constant alcohol or substance abuse, significant use of possible or known cognition-impairing drugs in the past 4 weeks and severe visual, hearing, mobility or motor coordination impairment. Excluded those participants who did not return for a second visit. | The Montreal Cognitive Assessment-Philippines (MoCAP) test was administered to detect cognitive impairment using a cut-off score of ≤21. | DR was assessed by funduscopic examination carried out through dilated pupils by an ophthalmologist and was classified according to the Early Treatment for Diabetic Retinopathy Study and International Clinical Diabetic Retinopathy Disease Severity Scales. Median (IQR) duration of DM 11.5 (12) years. | Prevalence of DR among cognitive impairment, 2016-2017. | DR was not associated with cognitive impairment, unadjusted OR 1.45 (0.68 to 3.11). |
| Poland, 2014, 2016, Gorska-Ciebiada^56, 57H, P, X^ | Aged ≥65, diagnosed with T2DM for one year and those who can follow study procedures. Excluded those who were diagnosed as depression or dementia, use of drugs which can affect cognition in the past 3 months, known neoplasm, constant alcohol or substance abuse, severe visual, mobility and motor coordination impairment, head trauma and major neurological or psychiatric diseases. | 2006 European Alzheimer’s Disease Consortium (cited Petersen 2004). | - | Prevalence of DR among cognitive impairment. | DR was associated with MCI unadjusted OR 2.25 (1.7 to 2.96) |
| Singapore, 2013, Ong^4P, P, X^ | Malays aged 40-79 living in designated study areas in southwestern Singapore, but only those aged ≥60 community-dwelling people were included in the current analyses. | Cognitive dysfunction was defined as an Abbreviated Mental Test score ≤6 of 10 for those with 0 to 6 years of formal education, and ≤8 for those with >6 years of formal education. | AMD was graded from retinal photographs according to the Wisconsin Age-Related Maculopathy Grading system. | Prevalence of AMD among cognitive impairment, Singapore Malay Eye Study (SiMES). | No association between AMD and cognitive impairment in unadjusted analysis (p=0.799) |
| Spain, 2019, Marquié^10C, P, X^ | Aged ≥50, presence of a consensus-based clinical diagnosis about the participants’ cognitive status and ability to complete the full ophthalmological exam and OCT scan. Excluded patients with severe dementia stages, equivalent to a Global Deteriorating Scale score >6. | Petersen’s criteria for MCI. DSM-V for dementia. | Age-Macular Degeneration Preferred Practice Patterns guidelines from the American Academy of Ophthalmology 2015, which uses the classification of the Age-Related Eye Disease Study and a more recent classification to define the early and intermediate stages of AMD. Glaucoma was based on the image of the head of the optic nerve and intraocular pressure measurements. | Prevalence of AMD or glaucoma among cognitive impairment or dementia, 2017-2018, NeuroOphthalmology Research At Fundació ACE (NORFACE) cohort. | - |
| Taiwan, 2015a, Chung^11P, R^ | Diagnosed with dementia ≥twice during the recruiting period with ≥one diagnosis made by a certified neurologist. Excluded those aged <40, a history of major psychosis or a substance-related disorder prior to the first dementia diagnosis. | ICD-9-CM codes 290.0-290.4, 294.1, 331.0-331.2, or 331.82. | ≥2 diagnoses of neovascular/wet AMD (ICD-9CM codes 362.42, 362.43, 362.52, or 362.53) prior to the first dementia diagnosis. | Prevalence of AMD among dementia, 2002-2011, AMD diagnosed before the diagnosis of dementia. | People with dementia were more likely to have had prior neovascular/wet AMD than controls adjusted OR 1.37 (1.14 to 1.65). |
| Taiwan, 2015b, Chung^25P, R^ | Received a first-time diagnosis of dementia (ICD-9-CM codes 290.0–290.3, 294.1, 331.0–331.2, or 331.82) during ambulatory care visits, received a diagnosis of dementia ≥twice coded in their ambulatory care claims, with ≥one being made by a certified neurologist or psychiatrist. Excluded those aged <45, those ever received a diagnosis of major psychosis or a substance-related disorder, stroke, or traumatic brain injury before the index date. | ICD-9-CM codes 290.0-290.3, 294.1, 331.0-331.2, or 331.82, diagnosis of dementia ≥twice coded in ambulatory care claims, with ≥1 diagnosis being made by a certified neurologist or psychiatrist. | ICD-9-CM codes 365.1, 365.10, or 365.11 and received treatment with topical antiglaucoma medication. POAG. | Prevalence of glaucoma among dementia, 2005-2011, glaucoma diagnosed before the diagnosis of dementia. | People with dementia were more likely to have had prior POAG than controls adjusted OR 1.44 (1.12 to 1.85). |
| Taiwan, 2017, Lai^26P, R^ | Aged ≥65 with new diagnosis of AD. | ICD-9 code 331.0. | ICD-9 codes 365.1 and 365.2. POAG and PACG. | Prevalence of glaucoma among dementia, 2000-2011, glaucoma diagnosed before the diagnosis of dementia. | The adjusted OR of AD was 1.50 in subjects with glaucoma (1.19 to 1.89), compared to subjects without glaucoma. |
| UK, 2014, Keenan^18P, R^ | Aged ≥50, admitted to a hospital with a diagnosis of dementia during the study period and no AMD before or at the time of recruitment. | - | In most cases patients undergoing admission for intravitreal anti-VEGF therapy, neovascular AMD (rather than geographic atrophy or early AMD). | Cumulative incidence of AMD among dementia, 1999-2011, ≥1 year. | The likelihood of being admitted for AMD following dementia was very low: the rate ratio was 0.07 (0.04 to 0.11). |
| UK, 2014, Williams^3H, P, X^ | Aged ≥65. | AD was defined by NINCDS criteria applied by dementia specialists as part of routine clinical care. MMSE: mean±SD 17.9±6.6. | The AMD grading system (referred to in this study as the “Whitla grades”) borrowed grade definitions employed in the Rotterdam Study. | Prevalence of AMD among dementia. | No AMD grade significantly influenced risk of AD after controlling for covariates. Compared to grade 0, ORs are grade 1 0.65 (0.4 to 1.1), grade 2 1.00 (0.5 to 1.9), grade 3 1.38 (0.6 to 3.2) |
| UK, 2015, Keenan^27P, R^ | Aged ≥55, admitted to a hospital with the first time diagnosis of AD or VaD during the study period and no POAG before or at the time of recruitment. | AD and VaD based on ICD-10. | ICD-10. POAG. | Cumulative incidence of glaucoma among dementia, 1999-2011, ≥ 1 year. | The likelihood of a hospital record of POAG following AD or VaD was very low, with rate ratios 0.28 (0.24 to 0.31) and 0.32 (0.28 to 0.37), respectively. |
| UK, 2016, Naidu^44P, P, X^ | Aged 18-75, diagnosed with T2DM in the last 6 months. Excluded those with DM other than T2DM, temporary residence and/or residence outside the catchment area, lack of fluency in English, movement from another primary care team, a terminal or separate advanced condition, severe mental illness (dementia, substance dependence, bipolar disorder, personality disorder), or severe advanced complications of DM (blindness, requiring dialysis or having undergone an above-the-knee amputation). | Lowest 10% of the sample distribution (score ≤17) on the modified Telephone Interview for Cognitive Status. | Defined as any retinal hemorrhage or microaneurysm found in either eye by an ophthalmologist. | Prevalence of DR among cognitive impairment, South London Diabetes Study (SOUL-D). | In a cohort of newly diagnosed T2DM, 22.4% of those with cognitive impairment and 11.9% of those without cognitive impairment had DR (p=0.11). |
| USA, 1986, Chandra^40P, R, X^ | People who had senile or pre-senile dementia as underlying, immediate, associated, or contributory cause of death. | ICDA 8, rubric 290. | - | Prevalence of glaucoma among dementia, 1978. | Glaucoma appeared more frequently (unadjusted OR 2.6) on the death certificates of AD cases than on those of controls. |
| USA, 2019, Smilnak^14H, R, X^ | Pathologic specimens of eyes and brains of autopsy subjects aged ≥75. | AD was graded in brain specimens by neuropathologists as previously described by Braak H and Braak E and in accordance with the NIA-AA and the CERAD guidelines. AD was defined as Stage III-VI with “moderate” or “frequent” neurotic plaque density, representing CERAD 2 or 3 scores. AD cases were further grouped into the categories of “early AD” (Braak and Braak Stages III-IV) and “late AD” (Braak and Braak Stages V-VI). Braak Stage III (n=42), IV (n=30), V (n=31), and VI (n=12). | Sarks stages. AMD severity was graded in a minimum of 5 eye sections per eye by a board-certified ophthalmic pathologist as previously detailed by Sarks. For cases with discrepant stages between the two eyes, the higher Sarks score for AMD was considered in the analysis. Sarks stages I (normal) and II (age-related changes): eyes without AMD (controls), stages III-IV: intermediate AMD, and stages V-VI: severe AMD due to the presence of either geographic atrophy or neovascular disease. The histopathologic diagnosis of advanced glaucoma was made when the following were observed: sparse retinal ganglion cells, diminished size of optic nerve axon bundles, and fibrotic thickening or “cupping” of the optic nerve. | Prevalence of AMD or glaucoma among dementia. | The rate of AMD was not significantly different between AD cases (53%) and controls (59.6%) (p=0.794). Comorbidity rates of advanced glaucoma, the only stage of glaucoma that can be reliability diagnosed via histopathology analysis, were similar between AD cases (13.9%) and controls (17.5%, p=0.412). |

**^a,b,c^**Presented as country/region, year, last name of the first author. ^a^Case selection: H hospital-based, C community-based, P population-based; ^b^Recruitment: P prospective, R retrospective; ^c^X representing cross-sectional data collection. Refer to Appendix C for references.

AD denotes Alzheimer’s disease, AMD age-related macular degeneration, CERAD Consortium to Establish a Registry for Alzheimer’s Disease, CI confidence interval, DM diabetes mellitus, DR diabetic retinopathy, ICD-10 International Classification of Diseases 10th revision, ICD-9-CM International Classification of Diseases ninth revision clinical modification, ICDA-8 International Classification of Diseases adapted eighth revision, IQR interquartile range, MCI mild cognitive impairment, MMSE Mini-Mental State Exam, NIA-AA National Institute of Aging and Alzheimer’s Association, NINCDS-ADRDA National Institute of Neurological and Communicative Disorders and Stroke-Alzheimer’s Disease and Related Disorders Association Work Group, NINDS-AIREN National Institute of Neurological Disorders and Stroke, and of the Association Internationale pour la Recherche et l’Enseignement en Neurosciences Work Group, OCT optical coherence tomography, OR odds ratio, PACG primary angle-closure glaucoma, POAG primary open angle glaucoma, SD standard deviation, T1DM type 1 diabetes mellitus, T2DM type 2 diabetes mellitus, UK United Kingdom, USA United States of America, VaD vascular dementia.

# eTable 3 Characteristics of studies on the frequency of cognitive impairment or dementia among people with age-related macular degeneration, glaucoma, or diabetic retinopathy

| **Source** | **Inclusion and exclusion criteria** | **Cognitive impairment or dementia diagnostic criteria, type, and severity** | **AMD, glaucoma or DR diagnostic criteria, type, and stage** | **Subgroup, study name, recruitment period, average length of follow-up (mean±SD or median (Q1, Q3))** | **Association between AMD, glaucoma, or DR and cognitive impairment or dementia (95% CI)** |
| --- | --- | --- | --- | --- | --- |
| Australia, 2006, Pham^5P, P, X^ | Aged ≥49, and participated in detailed examinations. | Cognitive impairment defined as an MMSE score ≤23. Mean MMSE 27.8 among people with early (n=273) and late (n=50) AMD. | AMD lesions were assessed from retinal photographs using the Wisconsin AMD Grading System. Early AMD (either large indistinct soft drusen or reticular drusen, or both large distinct soft drusen and retinal pigment epithelial abnormalities, hyperpigmentation or hypopigmentation, within the superimposed grading grid), late AMD (both geographic atrophy and neovasuclar maculopathy). | Prevalence of cognitive impairment among AMD, Blue Mountains Eye Study, 1992-1994. | Late AMD was associated with cognitive impairment adjusted OR 3.7 (1.3 to 10.6). No significant association was found between cognitive impairment and early AMD, adjusted OR 1.4 (0.8 to 2.6). |
| Canada, 2015, Harrabi^16H, P, X^ | Aged ≥65, for AMD cases bilateral AMD and visual acuity of worse than 20/40 in their better eye, for glaucoma cases bilateral glaucoma and a visual field mean deviation worse than or equal to 4dB in the worse eye. Excluded those with ≥two of AMD, glaucoma and Fuch’s corneal dystrophy. | Participants with scores <17 on MMSE blind version met the criteria for cognitive impairment. | - | Prevalence of cognitive impairment among AMD or glaucoma, 2009-2013. | A greater percentage of people in the groups with eye diseases (among AMD 14%, among glaucoma 12%) had cognitive impairment compared with the control group (3%). |
| China, 2020, Yu^45H, P, X^ | T2DM patients with DR who were treated at the recruitment site during the recruitment period. Excluded those with acute inflammatory diseases and autoimmune disease, acute complications of DM, neurological diseases that cause cognitive impairment, other ocular conditions that affect visual function, patients with lung cancer, liver dysfunction, cardiac and respiratory failure, patients with alcoholism and drug abuse, and patients with incomplete information. | MCI diagnostic guidelines developed by the National Institute on Aging and the Alzheimer’s Association workgroups: a decline in cognition corroborated by an informant or a clinician, objective evidence of impairment in ≥one cognitive domains, assessed by MMSE, preservation of independence in daily life, not demented. Cognitive impairment identified as an MMSE score >23 and <27. | Each subject was examined by a clinical ophthalmologist with the use of an ophthalmoscope. The diagnosis and staging of DR were defined by fluorescein fundus angiography. PDR (n=44). | Prevalence of cognitive impairment among DR, 2018-2019. | - |
| Denmark, 2007, Kessing^28P, R^ | - | ICD-8 and ICD-10 for AD and other dementia. | ICD-8 and ICD-10. PACG and POAG. | Accumulative incidence of dementia among glaucoma, 1977-2001 inpatient registries and 1994-2001 outpatient registries, 4.1 (1.6, 7.9). | Patients with POAG did not have increased rate of subsequent dementia compared to the general population, RR 1.08 (0.97 to 1.20). |
| Denmark, 2012, Bach-Holm^23H, R^ | All patients diagnosed with unilateral or bilateral NTG during the study period at the recruitment center. | ICD-8 and ICD-10. | The NTG diagnostic criteria were verified glaucomatous visual field defect, glaucomatous optic disc and diurnal curves (8 and 12 am, 4 and 7 pm) with peak pressure ≤24 mmHg after a 3-week wash out of any existing medication. | Accumulative incidence dementia among glaucoma, 1980-2001, 12.7 years. | Observed number of dementia cases/expected number of dementia cases: 0.2 (0.01 to 1.11). NTG was not associated with an increased risk of developing dementia/AD. |
| Japan, 2017, Honjo^41H, P, X^ | Aged ≥75 and no history of dementia, glaucoma was the only cause of visual field damage, axial length between 26 and 21 mm, patients underwent ≥3 visual field tests prior to the current study, and logMAR visual acuity ≤0.5. Excluded those with unreliable visual fields defined as any of the fixation loss, false positive, or false negative >33%. | MMSE score ≤23 points defined as severe to moderate cognitive impairment, MMSE score 24 to 27 points defined as MCI. | - | Prevalence of cognitive impairment among glaucoma, 2015. | - |
| Malaysia, 2019, Raman^21H, P^ | Aged ≥50, BCVA (6/12) in the better eye, no scotoma in the central 10 degree field of visual field. Excluded patients with ocular conditions, e.g. macular degeneration, media opacity, and dementia or other neurological diseases. | ≤3 on clock drawing test. “Cognitive impairment”. | The Hodapp-Parrish-Anderson Glaucoma Grading Scale to grade glaucoma severity, but definition/diagnostic criteria not reported. Early glaucoma stage 48.6%, moderate 31.2%, and severe 20.2%. | Prevalence of cognitive impairment among glaucoma, 2017, 4.4±0.8. | - |
| Netherlands, 1999, Klaver^2P, P^ | Aged ≥75. Excluded those diagnosed with dementia at baseline. | A three-step protocol MMSE and the Geriatric Mental State schedule, the CAMDEX diagnostic interview, and examination by a neurologist. AD based on NINCDS-ADRDA criteria. | Grading of fundus transparencies according to the international classification system. Stage 1, the presence of only soft distinct druse of >63μm in the absence of pigmentary irregularities and atrophic or endovascular macular degeneration; stage 2, the presence of either distinct drusen with pigmentary irregularities or indistinct or reticular drusen; stage 3, the presence of indistinct or reticular drusen with pigmentary irregularities; and stage 4, the presence of either atrophic or neovascular end-stage macular degeneration. | Accumulative incidence of dementia among AMD, the Rotterdam Study, 1990-1993, 2.1 years (mean). | Subjects with advanced AMD at baseline showed an increased risk of incident AD RR 2.1 (1.1 to 4.3) adjusted for age and gender, but this risk decreased after additional adjustment for smoking and atherosclerosis RR 1.5 (0.6 to 3.5). |
| Norway, 2004, Ritland^42H, R^ | Patients with POAG who were ultimately hospitalized at the recruiting site. Excluded those with DM. | - | POAG. | Prevalence of dementia among glaucoma, 1961-1970, follow-up to Apr 1, 1994. | - |
| Singapore, 2012, Ong^7P, P, X^ | Aged 60-80, agreed to participate. Excluded those who did not have cognitive test data. | Abbreviated Mental Test score of ≤6 (out of 10, 0 to 6 years of formal education), an AMT score of ≤8 (>6 years of formal education). | AMD graded from retinal photographs according to the Wisconsin Age-Related Maculopathy Grading System. International Society Geographical and Epidemiological Ophthalmology scheme based on gonioscopy, optic disc characteristics, and/or visual fields results. | Prevalence of cognitive impairment among AMD or glaucoma, Singapore Malay Eye Study, 2004-2006. | No significant independent associations were observed between AMD or glaucoma and cognitive impairment: adjusted OR 1.36 (0.34 to 5.45) and 2.09 (0.52 to 8.31) respectively. |
| Singapore, 2019, Gupta^49P, P^ | Aged ≥60, T1DM or T2DM, gradable fundus photos at baseline, no cognitive impairment at baseline, attended 6-year follow up visit, had information on risk factors. | Cognitive impairment based on the score from Abbreviated Mental Test ≤6 for education level between 0-6 years and ≤8 for education level >6 years. | Modified Airlie House classification system: none DR ETDRS (level 10), minimal/mild (level 20-35) and moderate or worse DR (level 43-90). Minimal or mild DR (n=142), moderate or worse DR (n=57). | Accumulative incidence of cognitive impairment among DR, the Singapore Epidemiology Eye Disease (SEED-1 and SEED-2) programme, 2004-2011, follow-up conducted between 2010-2016. | Compared to participants without DR, those with any DR had increased risk of incident cognitive impairment adjusted OR 2.32 (1.07 to 5.03), those with moderate or worse DR had an increased risk, adjusted OR 3.41 (1.06 to 11.00). |
| South Korea, 2012, Woo^8H, P, X^ | All patients who had AMD categorized as ≥3 according to the Age-Related Eye Disease Study classification system. Excluded those with cognitive disorders such as dementia, major psychiatric disorders such as major depressive disorders (according to the DSM-IV criteria), or other severe medical conditions that may affect cognition. | MCI was defined according to the Revised Diagnostic Criteria, proposed by the International Working Group on MCI. MMSE 24.97±3.30. | AMD categorized as ≥3 according to the Age-Related Eye Disease Study classification system: early AMD, presence of ≥1 large drusen, numerous medium-sized drusen, or geographic atrophy that does not extend to the center of the macula, late exudative AMD, choroidal neovascularization or any of its potential sequelae including a fibrotic scar, late non-exudative AMD or geographic atrophy, geographic atrophy extending to the center of the macula. Early n=46, exudative n=107 (non-polypoidal choroidal vasculopathy n=82 and polypoidal choroidal vasculopathy n=25), geographic atrophy n=17. | Prevalence of cognitive impairment among AMD, 2008-2010. | The rate of MCI was higher in people with AMD than in controls (52.4% vs 26.8%, p<0.001), adjusted OR 3.127 (1.855 to 5.271). |
| South Korea, 2018, Moon^37P, R^ | POAG, Korean Classification of Diseases code H401, as the main diagnosis code, having a visual field test code (E6691), and prescribed glaucoma medication. To exclude chronic POAG patients, we included patients who were diagnosed with POAG from 2004 to 2008. Excluded patients who were diagnosed with AD before the POAG diagnosis. | Korean Classification of Diseases codes F009, G300, G301, G308, and G309. AD. | Korean Classification of Diseases code H401, POAG. | Accumulative incidence of dementia among glaucoma, 2002, 6.99 (median). | POAG was significantly associated with an increased incidence of AD adjusted HR 1.403 (1.180 to 1.669). |
| South Korea, 2020, Choi^12P, R^ | Diagnosed with AMD in 2003-2005, aged ≥50. Excluded those diagnosed with AMD during 2002, with missing values on covariates, diagnosed with AD prior to the index date (January 1, 2006), or died before the index date. | Being prescribed anti-dementia drugs (donepezil, galantamine, rivastigmine or memantine) under the ICD-10 code for AD (F00, G30). | ICD-10 H35.3. | Accumulative incidence of dementia among AMD, 2003-2005, follow-up 2006-2013. | Compared to non-AMD participants, participants with AMD had higher risk for AD, adjusted HR 1.48, (1.25 to 1.74). |
| Taiwan, 2010, Lin^29P, R, X^ | Aged ≥40, with ≥3 consensus OAG diagnoses. Excluded patients who were not prescribed topical anti-glaucoma medication and were not operated on for glaucoma during the study period, and patients who had ever undergone laser iridotomy or had never undergone a visual field examination in the previous 5 years. | ICD-9, dementia were counted if claims occurred in an inpatient setting or in >2 ambulatory care. | ICD-9 codes 365.1-365.11 POAG. | Prevalence of dementia among glaucoma, 2005. | People with OAG were more likely to have dementia, adjusted OR 1.19 (1.09 to 1.29), than those without OAG. |
| Taiwan, 2014, Lin^30P, R^ | Aged ≥65, ≥2 visits with POAG diagnosis one-month apart, received anti-glaucoma medication or glaucoma surgery and visited ≥once to an eye clinic or a hospital based ophthalmology department during the study period. Excluded patients with a history of AD, other forms of dementia, or Parkinson’s disease, or who had received a prescription of acetylcholinesterase inhibitors or an anticholinergic agent and dopaminergic agent. | AD (ICD-9-CM 331.0), other forms of dementia (ICD-9-CM 290, 290.0-4, and 290.8-9). | ICD-9-CM codes 365.10 open angle glaucoma unspecified, 365.11 POAG, 365.12 NTG. | Accumulative incidence of dementia among glaucoma, 2001-2008, follow-up ranged from 1 to 8 years. | People with POAG had a higher risk of AD than the controls did, adjusted HR 1.40 (1.03 to 1.90, p=0.033). |
| Taiwan, 2015, Tsai^13P, R^ | Aged ≥65 and had ≥2 clinical visits with a diagnosis of AMD. Excluded those who had been diagnosed with AMD before 2001, diagnosed as AD (ICD-9-CM 331.0) or dementia (ICD-9-CM 290.xx) before enrolment, and a history of stroke (ICD-9-CM codes 430-438). | AD (ICD-9-CM 331.0) or senile dementia (290.0, 290.2, 290.20, 290.21, 290.3). | ICD-9-CM code 362.50, 362.51, and/or 362.52. | Accumulative Incidence of dementia among AMD, 2001-2009, 4.2±2.5. | The incidence of AD or senile dementia was higher in people with AMD than in the controls, adjusted HR 1.44 (1.26 to 1.64). |
| Taiwan, 2016, Su^31P, R^ | Newly diagnosed with glaucoma during the recruiting period. | Dementia (ICD-9-CM 290, 294.1, and 331.0). | POAG (ICD-9-CM 365.1) and PACG (ICD-9-CM 365.2). | Accumulative incidence of dementia among glaucoma, 2000-2009, 6.3 years (mean) for POAG and of 6.4 years (mean) for PACG. | People with glaucoma had a significant higher risk of dementia than the those without glaucoma, adjusted HR 1.13 (1.01 to 1.27). |
| Taiwan, 2017, Lai^33P, R^ | Aged ≥65 with new diagnosis of glaucoma. To enhance the diagnosis validity, subjects who had the same diagnosis in 3 consecutive clinical records during the study period were included. | ICD-9. | ICD-9 codes 365.1 and 365.2. POAG (n=1956) and PACG (n=2374). | Prevalence of dementia among glaucoma, 2000-2010, glaucoma diagnosed before the diagnosis of dementia. | The glaucoma group had higher proportions of dementia than the non-glaucoma group, p<0.05. |
| Taiwan, 2018, Chen^32P, R^ | Patients with newly diagnosed NTG. Excluded patients with NTG diagnosed from 1 January 1996 to 31 December 2000, and subjects who received a diagnosis of AD or dementia before the date of the first NTG claim or enrolment. | ICD-9-CM codes 331.0. AD. | NTG (ICD-9-CM codes 365.12). | Accumulative incidence of dementia among glaucoma, 2001-2013, 4.92±3.29. | NTG group had a significantly higher risk of AD, adjusted HR 1.52 (1.41 to 1.63). |
| Taiwan, 2019, Chen^34P, R^ | Aged >20 with a diagnosis of PACG. Excluded patients with a history of POAG diagnosed before date of diagnosis of PACG and/or with juvenile and congenital glaucoma. | ICD-9-CM code 290, 294.1 and 331.0. | ICD-9-CM code 365.2. PACG. | Prevalence of dementia among glaucoma, 2005-2011, dementia diagnosed before the diagnosis of glaucoma. | Dementia was not associated with PACG, unadjusted OR 0.98 (0.79 to 1.21). |
| Taiwan, 2020, Kuo^35P, R^ | A diagnosis of glaucoma according to the ICD-9 or ICD-10 codes, a diagnosis of glaucoma made by an ophthalmologist, receipt of an OCT exam or visual field test before the glaucoma diagnosis, and aged 20-100. Excluded those who had blindness, an ophthalmic malignancy, eyeball removal surgery, severe ocular trauma, or death before the index date. | ICD-9/10 codes for AD, VaD and Parkinson’s disease dementia. | POAG (including normal tension) and PACG (ICD-9/10). | Accumulative incidence of dementia among glaucoma, 1997-2016, 5 years (mean). | There was no difference in the rate of dementia, adjusted HR 0.961 (0.886 to 1.043, p=0.3443), between subjects with glaucoma and the controls. Compared to the controls, different severities of glaucoma did not alter the incidence of subsequent dementia. |
| Turkey, 2014, Dag^17H, P, X^ | Aged ≥60, bilateral AMD, and BCVA no worse than 2/10. Excluded those with glaucoma, diabetic retinopathy, or cataract surgery within 6 months, dementia, and presence of life threatening illness. | <21 on MMSE as cut offs for cognitive impairment. Also, used <21 on MoCA as cut offs for cognitive impairment. | - | Prevalence of cognitive impairment among AMD, 2012. | - |
| Turkey, 2015, Seden^9H, P, X^ | Aged ≥65, no AD, and sufficient visual acuity (≥20/100). Excluded those with neurological and psychiatric diseases or any other medical conditions that might affect cognition. | NINCDS-ADRDA. MMSE mean±SD 24.3±3.88. | Criteria proposed by the Age- Related Eye Disease Study. The AMD patients were classified as follows: 1) early AMD: presence of a few (<20) medium-sized drusen or retinal pigmentary abnormalities; 2) intermediate AMD: at least 1 large drusen, numerous medium-sized drusen, or geographic atrophy that does not extend to the center of the macula; 3) late dry (nonvascular) AMD (d-AMD): drusen and geographic atrophy extending to the center of the macula; 4) late wet (vascular) AMD (w-AMD): choroidal and retinal neovascularization and its sequelae, such as sub-retinal fluid, lipid deposition, hemorrhage, retinal pigment epithelium detachment, and fibrotic scar. The eyes with lower BCVA were used in the statistical analysis. | Prevalence of dementia among AMD | The frequency of AD was higher in people with AMD (40.7% in AMD and 20.4% in control group, p =0.03). |
| Turkey, 2015, Ogurel^46H, P, X^ | Excluded those with history of ocular trauma, ocular surgery within 6 months, BCVA worse than 1/10, history of cerebrovascular events, the presence of life threatening illness and dementia. | <21 on the MoCA was used for “cognitive impairment”. | Early Treatment of Diabetic Retinopathy Study classification. Mild, severe NPDR and PDR. | Prevalence of cognitive impairment among DR, 2014. | - |
| UK, 2013, Crosby-Nwaobi^50C, P, X^ | Diagnosis of T2DM for ≥5 years, aged >30, and presence of confirmed PDR. Excluded those with severe mental illness, terminal illness, or stroke determined from their medical notes, any other form of DM including starting insulin within 1 year of diagnosis, non-assessable fundus photographs, unable to converse in English, and severe visual impairment with a bilateral BCVA of 1.0 (logMAR) at 1 m or counting fingers, hand movements, and perception or no perception of light. | Cognitive impairment defined as 19≤MMSE≤24. Dementia defined as Mini-Cog 0 to 2. | Early Treatment of Diabetic Retinopathy Study grading criteria (PDR ≥level 61). PDR. | Prevalence of cognitive impairment or dementia among DR, the South East London Diabetic Retinopathy Study. | The MMSE cutoff scores showed that 12% of the no/mild DR group (n=31) had positive screening results for dementia or significant cognitive impairment compared with 5% in the PDR group (n=6, p=0.035). |
| UK, 2014, Keenan^18P, R^ | Aged ≥50, admitted to a hospital with a diagnosis of AMD during the study period and no dementia before or at the time of recruitment. | - | In most cases patients undergoing admission for intravitreal anti-VEGF therapy, neovascular AMD (rather than geographic atrophy or early AMD). | Accumulative incidence of dementia among AMD, 1999 to 2011, ≥1 year. | The risk of dementia following AMD was not elevated. The rate ratio was 0.91 (0.79 to 1.04). |
| UK, 2015, Keenan^27P, R^ | Aged ≥55, admitted to a hospital with the first time diagnosis of POAG during the study period and no dementia before or at the time of recruitment. | AD and VaD based on ICD-10. | ICD-10. POAG. | Accumulative incidence of dementia among glaucoma, 1999-2011, ≥1 year. | The risk of AD following a diagnosis of POAG was not elevated: the rate ratio was 1.01 (0.96 to 1.06). The risk of VaD after POAG was modestly elevated, with rate ratio 1.10 (1.05 to 1.16). |
| USA, 2009, Baker^6P, P, X^ | People living in the household of each individual sampled from the Medicare eligibility list of Health Care Financing Administration sampling frame, aged ≥65 at the time of the examination, non-institutionalized, expected to remain in the area for the next 3 years, be able to give informed consent, did not require a proxy respondent. Excluded those home-bound, receiving hospice treatment, radiation therapy or chemotherapy for cancer (not including skin cancer), unable to communicate with the study interviewer at baseline, unavailable or ungradable retinal photographs, invalid cognitive function test, taking antipsychotic or antidepressant agents, and a history of stroke at follow-up visit during 1997 and 1998. | Dementia was defined as a progressive or static cognitive deficit of sufficient severity to affect the subjects’ activities of daily living and a history of normal intellectual function before the onset of cognitive abnormalities, and also impairments in two cognitive domains (DSM-IV). | Modified Wisconsin AMD grading system (superimposition of a circular grid of the retinal photograph). AMD (n=351): early (n=324) and late AMD (n=27), prevalence of dementia, etc was reported for 222 participants with early AMD only. | Prevalence of dementia among AMD, the Cardiovascular Health Study, 1989-1993, retinal photographs taken in 1997-1998 and dementia assessed in 1998-1999. | No association of early AMD with dementia or AD: adjusted OR 0.98 (0.57 to 1.69) for dementia; adjusted OR 1.09 (0.57 to 2.08) for AD. |
| USA, 2011, Whitson^19H, P^ | Aged ≥65 with AMD. Excluded those with hearing problem and/or language barriers. | TICS-m administered in-person. Scores on this 50-point test were adjusted for years of education. Cognitive status was classified as normal (scores >30), marginal impairment (28-30), or cognitive impairment (≤27). | Wet AMD (n=51) dry AMD (n=19) | Prevalence of cognitive impairment among AMD, 2007-2008, 115±29 days and maximum follow-up 297 days. | - |
| USA, 2012, Ou^36P, R^ | Aged ≥68, no AD or any other dementia at baseline, and had a diagnosis of OAG at two claims, or one claim for OAG diagnosis and one claim for OAG related procedure. Participants also had to be present in the system at least another year after January 2014 and ≥3 visits for healthcare during the follow-up period. Excluded those aged <68 or >95, no 3 year full look-back period and residing outside of USA for >12 months during 3 years before the recruitment period. | AD (ICD-9-CM 331.0) or other dementia (ICD-9-CM 290, 290.0-4, 290.8-9, 291.0, 291.2, 292.82, 294.0-1, 294.8, 331.1-2, 331.7, 331.82, 797.xx). | ICD-9-CM codes 365.1 OAG, 365.10 OAG, unspecified, 365.11 POAG; 365.12 low tension glaucoma; 365.15 residual stage of OAG, or one diagnosis of OAG and one OAG-related procedure (CPT-4 codes 65855, 66150, 66155, 66160, 66165, 66170, 66172, 66180, 66185, 66250, 66710, 66711, 66720). | Accumulative incidence of dementia among glaucoma, 1991 to 1993, 14-year follow-up. | Individuals with OAG have a decreased rate of AD or other dementia diagnosis compared to control patients without an OAG diagnosis, adjusted HR 0.93 (0.91 to 0.95). |
| USA, 2014, Exalto^51P, R^ | Alive at baseline (1 Jan 1998) without dementia diagnosis, no gap of ≥3 months in health plan membership during the 2 years prior to baseline, aged ≥60 at baseline. To restrict false positive diagnosis of DR, only the severe sight threatening DR forms (PDR and diabetic macular edema) were included. | - | PDR and/or diabetic macular edema identified in 1996-1998 using either records for inpatient treatment for PDR: panretinal photocoagulation (CTP4 code 67228); for diabetic macular edema: focal and grid photocoagulation (CTP4 codes 67208 67210); or outpatient diagnoses made in ophthalmology (ICD 9 codes 250.5+362.02 for PDR; ICD 9 codes 250.5+362.53 or 250.5+362.83 for diabetic macular edema). | Accumulative incidence of dementia among DR, 1994 to 1997 and 1996 to 1998, 6.6 years (mean). | Diabetic patients with severe diabetic retinal disease (i.e., PDR or diabetic macular edema) have an increased risk of dementia compared to diabetic patients without severe diabetic retinal disease, adjusted HR 1.32 (1.17, 1.49). |
| USA, 2018, Rodill^52P, R^ | Members of KPNC with T1DM, with no prevalent dementia diagnoses, and aged ≥50 at any time within the study period. Patients were considered if they fit all of the following criteria: a) had either two ICD-9 codes for T1DM without any T2DM codes or a ratio of T1DM:T2DM ICD-9 codes ≥0.75 in the Kaiser Permanente electronic database since start of membership; b) insulin prescriptions during the study period; and c) did not have prescriptions of any hypoglycemic agent other than insulin or metformin. Excluded those with ICD-9 coded AD (331.0), VaD (290.4x), nonspecific dementia (290.0, 290.1x, 290.2x, 290.3, 294.1x, 294.2x, and 294.8). Frontotemporal dementia (ICD-9 331.1x) and dementia with Lewy Bodies (ICD-9 331.82) were eligible but no cases were identified. | - | Proliferative DR (ICD-9 362.02; CPT-4 67228), macular edema (ICD-9 362.07, 362.53, 362.83; CPT-4 67208, 67210) or nonspecific DR (ICD-9 250.5x, 362.0x). | Accumulative incidence of dementia among DR, 1996-2015, 6.7±5.4. | No significant association was found between DR and incident dementia adjusted HR=1.12 (0.82 to 1.54). |
| USA, 2020, Schwaber^15H, R, X^ | Aged >75, underwent autopsy and a post-mortem interval of <72 h. | Braak and Braak staging used to characterize the severity of AD, stages 0, I, and II non-AD, stages III-IV early AD, stages V-VI late AD. “Non-AD dementia” if any other non-Alzheimer’s neuropathological degeneration, such as frontotemporal degeneration, Lewy bodies, Parkinson’s disease, and hippocampal sclerosis. | Sarks stages I (normal) and II (age-related changes) no AMD, stages III-IV intermediate AMD, stages V-VI severe AMD (presence of either geographic atrophy or neovascular disease). Both eyes were graded but only one eye was included in the analysis, if there was a discrepancy in the stage of the two eyes, and the higher score was used to classify the Sarks stage. | Prevalence of dementia among AMD, 1997-2017. | The prevalence of AD was lower in AMD subjects (63%) compared to non-AMD subjects (73%), even when grouped by severity (all p>0.15). The likelihood of AD was significantly less in AMD subjects, adjusted OR 0.46 (0.21 to 0.99), p=0.046. |

**^a,b,c^**Presented as country/region, year, last name of the first author. ^a^Case selection: H hospital-based, C community-based, P population-based; ^b^Recruitment: P prospective, R retrospective; ^c^X representing cross-sectional data collection. Refer to Appendix C for references.

AD denotes Alzheimer’s disease, AMD age-related macular degeneration, BCVA best corrected visual acuity, CAMDEX Cambridge mental disorder examination, CI confidence interval, DM diabetes mellitus, DR diabetic retinopathy, DSM-IV Diagnostic and Statistical Manual of Mental Disorders fourth edition, ETDRS Early Treatment of Diabetic Retinopathy Study, HR hazard ratio, ICD-10 International Classification of Diseases 10th revision, ICD-9-CM International Classification of Diseases ninth revision clinical modification, ICDA-8 International Classification of Diseases adapted eighth revision, KPNC Kaiser Permanente Northern California, logMAR logarithm of the min angle of resolution, MCI mild cognitive impairment, MMSE Mini-Mental State Exam, MoCA Montreal Cognitive Assessment, NINCDS-ADRDA National Institute of Neurological and Communicative Disorders and Stroke-Alzheimer’s Disease and Related Disorders Association Work Group, NTG normal tension glaucoma, OCT optical coherence tomography, OR odds ratio, PACG primary angle-closure glaucoma, PDR proliferative diabetic retinopathy, POAG primary open angle glaucoma, RR relative risk, SD standard deviation, T1DM type 1 diabetes mellitus, T2DM type 2 diabetes mellitus, TICS-m telephone interview for cognitive status modified, UK United Kingdom, USA United States of America, VaD vascular dementia.

# eTable 4a Quality assessment

| Country/region, year, last name of the first author, study | Australia, 2008, Bruce,^43^ the Fremantle Diabetes Study | Australia, 2014, Finger,^48^ Diabetes Management Project | Canada, 2014, Pelletier^38^ | China, 2020, Xia^53^ |
| --- | --- | --- | --- | --- |
| 1. Was the study’s target population a close representation of the national population with age-related macular degeneration, glaucoma, diabetic retinopathy, cognitive impairment, mild cognitive impairment or dementia in relation to relevant variables, e.g. age, sex? | High risk. Only included those who were aged ≥68, and had a diagnosis of open-angle glaucoma at two claims or a combined of one claim for open-angle glaucoma diagnosis and one claim for open-angle glaucoma related procedure. Participants also had to be present in the system at least another year after January 2014 and had at least three visits for healthcare during the follow-up period. | High risk. Included individuals with diabetes (type 1 or 2), ≥ 18 years old, with and without diabetic retinopathy, English-speaking, free of significant hearing and cognitive impairment, and living independently. | High risk. Excluded people with other form of dementia, massive stroke, and long-term use of corticosteroids. | High risk. Excluded those with any metabolic diseases that may affect cognitive function temporarily, such as acute carbohydrate metabolic events in the last 3 months, including severe hypoglycemia, diabetic ketoacidosis, diabetic hyperglycemia hypertonic coma, hypothyroidism; history of head trauma, cerebral intake, mental and neurological disorders such as depression, anxiety, delirium, severe lung or kidney diseases, heart failure, malignant tumors; history of drug dependence/abuse; used any of antidepressant drugs, anti-Parkinson drugs, anti-epileptic drugs, sedative hypnotic drugs, etc. in the last month; using cognitive dysfunction medications such as donepezil, memantine. |
| 1. Was the sampling frame a true or close representation of the target population? | Low risk. Population-based. | High risk. Hospital-based. | High risk. Hospital-based. | High risk. Hospital-based. |
| 1. Was some form of random selection used to select the sample, OR, was a census undertaken? | Low risk. All eligible cases included. | Low risk. All eligible cases were recruited. | Low risk. “Over the one year study period, 220 patients fulfilling our inclusion/exclusion criteria were admitted to the hospital and were diagnosed with Alzheimer’s Disease or mixed dementia.” | Low risk. All eligible cases were included. |
| 1. Was the likelihood of non-response bias and/or lost to follow-up minimal? | High risk. There may be survivor bias, as “There was considerable attrition from the Fremantle Diabetes Study; this left a study sample that was relatively healthy but resulted in a fairly small sample with relatively few cases of dementia.” | High risk. “There were four outcomes at the point of recruitment: the individual agreed to participate, declined to participate, considered the invitation with no definitive response or were missed by the recruiter”, but the numbers were not reported. | Low risk. Medical chart review, no need to take consent. | High risk. Non-response rate was not reported. |
| 1. Were data collected directly from the subjects or medical records (as opposed to a proxy)? | Low risk. “At baseline assessment between 1993 and 1996, a comprehensive sociodemographic and clinical history was taken, a physical examination was performed” and “Cognitive assessment was conducted in 2001 and 2002.” both directly with the participants. | Low risk. Collected from the subjects. | Low risk. Medical records should have been taken directly from the participants. | Low risk. A neurologist qualified after memory clinic training assessed the diagnosis of cognitive impairment and dementia. The study team accomplished laboratory inspection, and diabetic retinopathy diagnosis should be collected from medical records. |
| 1. Was an acceptable case definition used in the study? | Low risk. “Retinopathy was defined as any grade of retinopathy detected by direct and/or indirect ophthalmoscopy in one or both eyes and/or more detailed assessment by an ophthalmologist.” and “Clinical Dementia Rating scale rating of 0.5” was the criterion for Cognitive impairment without dementia | High risk. Modified Airlie House Classification System was used for diabetic retinopathy, but cut-off on a scale was used for cognitive impairment. | High risk. The definition or diagnostic criteria for glaucoma was not reported, though Diagnostic and Statistical Manual of Mental Disorders, fourth edition and recommendations from the National Institute on Aging-Alzheimer’s Association workgroups on diagnostic guidelines for Alzheimer’s disease were used to define dementia. | High risk. Core clinical criteria for mild cognitive impairment was from the National Institute of Aging and Alzheimer’s Association and Petersen’ criteria, but diabetic retinopathy diagnostic criteria unclear. |
| 1. Was the study instrument that measured the parameter of interest (i.e. age-related macular degeneration, glaucoma, diabetic retinopathy, cognitive impairment and dementia) shown to have reliability and validity? | High risk. Not sure about the validity of diabetic retinopathy diagnosis. | Low risk. Diabetic retinopathy was assessed by the investigators on the fundus photographs. | Low risk. “Patients with a presumed diagnosis of dementia are clinically evaluated to insure that the diagnosis is valid according to published criteria”. | High risk. Although “a neurologist who was qualified after the memory clinic training performed the cognitive assessment and verified the diagnosis of mild cognitive impairment and dementia”, it is unclear whether the diabetic retinopathy diagnosis was centrally adjudicated. |
| 1. Was the same mode of data collection used for all subjects? | Low risk. | Low risk. | Low risk. | Low risk. |
| 1. Were the numerator(s) and denominator(s) for the parameter of interest appropriate? | High risk. The numerator was not reported. | Low risk. | Low risk. | Low risk. |

# eTable 4b Quality assessment

| Country/region, year, last name of the first author, study | France, 2015, Verny,^54^ GERODIAB study | Germany, 2002, Bayer^22^ | Germany, 2019, Michalowsky^39^ | Greece, 2015, Tsolaki^20^ |
| --- | --- | --- | --- | --- |
| 1. Was the study’s target population a close representation of the national population with age-related macular degeneration, glaucoma, diabetic retinopathy, cognitive impairment, mild cognitive impairment or dementia in relation to relevant variables, e.g. age, sex? | High risk. Included those aged ≥ 70 years, activities of daily living score ≥3/6 and no concurrent acute conditions; excluded those with type 1 diabetes or secondary diabetes, loss of autonomy (activities of daily living score <3/6), acute disease stage. | High risk. The exclusion of the people with uncontrolled diabetes or Alzheimer’s disease associated with Parkinson’s disease has made the target population unrepresentative of the national population with dementia. | Low risk. No specific exclusion criteria. | High risk. Although there is no specific exclusion criteria, the inclusion criteria was unclear. |
| 1. Was the sampling frame a true or close representation of the target population? | Low risk. Community-based. | High risk. Nursing home-based. | Low risk. Community-based. | High risk. Hospital-based. |
| 1. Was some form of random selection used to select the sample, OR, was a census undertaken? | Low risk. “Consecutively” mentioned. | Low risk. “All institutionalized residents of the four nursing homes including the patients with Alzheimer’s disease”. | Low risk. All dementia cases by one of the 1203 general practitioners have been identified. | High risk. Unclear whether consecutive or random sampling. |
| 1. Was the likelihood of non-response bias and/or lost to follow-up minimal? | High risk. Non-response rate was not reported. | Low risk. Since this study was conducted through chart review, we assumed there was no non-response bias. | High risk. The diagnoses listed in the files of the general practitioners are usually used for reimbursement. Therefore, the data may not be complete, especially not for vision impairment diagnoses, which are, in most cases, not documented in general practitioners’ files. Among the elderly, the prevalence of vision impairment is approximately 70%. In this study, diagnoses related to vision impairment of any type was only documented in 28% of those with dementia, demonstrating that vision impairment was underdiagnosed and underrepresented in this dataset. | High risk. Response rate was not reported. |
| 1. Were data collected directly from the subjects or medical records (as opposed to a proxy)? | Low risk. Diabetes assessment and a geriatric assessment were systematically carried out. | Low risk. As this was chart review and “All patients were examined by one of the investigators with subspecialty training in glaucoma.”, we assumed this investigator had examined all the participants directly. | Low risk. Glaucoma diagnosis was collected from the medical records. | Low risk. “All participants were checked for dementia with the application of a neuropsychological battery consisting of the Mini Mental State Examination, the Functional Rating Scale for Symptoms of Delirium, the Neuropsychiatric Inventory, the Hindi Mental State Examination and the Geriatric Depression Scale” and “The ocular examination was based on visual acuity control (reading of an optotype from a distance of 5 meters), tonometry with a Goldmann’s applanation tonometer after application of a local anaesthetic (hydrochloric proxymetacaine 0.5%), and fundoscopy after the application of tropicamide solution 0.5% and of the a-adrenergic stimulator phenylefrine in a solution of 5%. The examination was concluded with a control of visual fields in a static perimetry apparatus type Octopus 900.” |
| 1. Was an acceptable case definition used in the study? | High risk. Cognitive disorders were defined by previously known dementia and/or Mini-Mental State Examination scores ≤24/30 and unclear about the diagnostic criteria for diabetic retinopathy. | Low risk. No problem with the diagnostic criteria for Alzheimer’s disease (National Institute of Neurological and Communicative Diseases and Stroke/Alzheimer’s Disease and Related Disorders Association) or primary open angle glaucoma (at least one of the two criteria: a characteristic pattern of glaucomatous visual field loss and a cup to disc ratio of ≥0.8 with an optic nerve head appearance consistent with glaucoma). | High risk. The criteria for dementia and for glaucoma were not specified. | Low risk. National Institute of Neurological and Communicative Diseases and Stroke/Alzheimer’s Disease and Related Disorders Association, clinical diagnostic criteria for dementia associated with Parkinson’s disease, consensus guidelines for the clinical and pathological diagnosis of dementia with Lewy bodies and frontotemporal lobar degeneration: a consensus on clinical diagnostic criteria and “Diagnostic criteria for glaucoma were the ones provided by the Thessaloniki Eye Study”. |
| 1. Was the study instrument that measured the parameter of interest (i.e. age-related macular degeneration, glaucoma, diabetic retinopathy, cognitive impairment and dementia) shown to have reliability and validity? | High risk. The diagnosis of diabetic retinopathy was not centrally adjudicated and unsure about its validity. | High risk. “Lack of objective optic disc photographs” and not centrally adjudicated. | High risk. Glaucoma diagnosis was recorded in the medical records using International Classification of Diseases codes and was not adjudicated by the investigators. | Low risk. The ocular examination was conducted by the study team. |
| 1. Was the same mode of data collection used for all subjects? | Low risk. | Low risk. | Low risk. | Low risk. |
| 1. Were the numerator(s) and denominator(s) for the parameter of interest appropriate? | High risk. The numerator (number of diabetic retinopathy cases) was not reported. | Low risk. | High risk. The number of glaucoma cases was not reported. | Low risk. |

# eTable 4c Quality assessment

| Country/region, year, last name of the first author, study | Italy, 2014, Mandas^1^ | Japan, 2006, Tamura^24^ | Japan, 2014, Sanke^55^ | Philippines, 2017, Blanquisco^47^ |
| --- | --- | --- | --- | --- |
| 1. Was the study’s target population a close representation of the national population with age-related macular degeneration, glaucoma, diabetic retinopathy, cognitive impairment, mild cognitive impairment or dementia in relation to relevant variables, e.g. age, sex? | High risk. Aged ≥65 years old. | Low risk. No specific exclusion criteria. | High risk. Excluded patients with severe infections within the preceding 2 weeks, scheduled for surgery or who had undergone surgery, with severe trauma, with psychiatric disorders, hypothyroidism, or brain tumors, with partial or complete olfactory dysfunction associated with sinusitis, allergic rhinitis, and deviated nasal septum, on steroid treatment, with an Mini-Mental State Exam score of <18. | High risk. People with diabetes but with neurocognitive, neurologic or psychiatric disorder, intracranial neoplasm, infectious disease, constant alcohol or substance abuse, significant use of possible or known cognition-impairing drugs in the past 4 weeks and severe visual impairment were excluded, which prevented the study “from including patients who were too sick, with unstable comorbidities and worse complications”. |
| 1. Was the sampling frame a true or close representation of the target population? | High risk. Hospital-based. | High risk. Hospital-based. | High risk. Hospital based. | High risk. Hospital-based. |
| 1. Was some form of random selection used to select the sample, OR, was a census undertaken? | Low risk. All eligible were recruited. | Low risk. Very likely all eligible were included. | Low risk. All eligible cases were included. | Low risk. “Consecutively” was mentioned. |
| 1. Was the likelihood of non-response bias and/or lost to follow-up minimal? | High risk. Informed consent was required, but unclear non-response rate and the characteristics of those who were included and excluded. | High risk. Non-response rate was not reported. | Low risk. All eligible cases agreed to participate. | High risk. Only 133 out of 226 elderly diabetics were included in the analyses, and there is no comparison of those who were included and excluded. |
| 1. Were data collected directly from the subjects or medical records (as opposed to a proxy)? | Low risk. “All subjects were screened at the Eye clinic, including ocular examination by slit lamp, visual acuity tests with Snellen chart, and intraocular pressure measurement with Goldmann applanation.” “Color fundus picture was taken in order to classify the presence of any sign of retinal disease.” “Detailed patient history was recorded, using a questionnaire focusing on individual medical history.” | Low risk. Alzheimer’s disease should have been diagnosed in the hospitals directly with the subjects. In addition, “These patients received ophthalmic examinations including estimation of width of the angle of the anterior chamber, and indirect ophthalmoscopy was performed in dilated pupils in all eyes to evaluate optic nerve head cup-to-disc ratios.” | Low risk. Collected from the subjects. | Low risk. Participants were interviewed and diabetic retinopathy was assessed by funduscopic examination. |
| 1. Was an acceptable case definition used in the study? | High risk. The diagnostic criteria for glaucoma was not reported, though “Possible/probable diagnosis of Alzheimer’s disease was made according to the criteria of the National Institute of Neurological and Communicative Disorders and Stroke-Alzheimer’s Disease and Related Disorders Association Work Group. Diagnosis of possible/probable vascular dementia was made according to the criteria of the National Institute of Neurological Disorders and Stroke, and of the Association Internationale pour la Recherche et l’Enseignement en Neurosciences Work Group (NINDS-AIREN).” | Low risk. The National Institute of Neurological and Communicative Disorders Association was used to diagnose Alzheimer’s disease, and “Probable open-angle glaucoma was diagnosed by width of the angle of the anterior chamber > grade 2 (method of Van Herick et al.), a vertical cup-to-disc ratio of the optic nerve head >0.7 and/or difference between the vertical cup-to-disc ratio in the eyes >0.2 with characteristic glaucomatous disc change.” | High risk. Not a proper diagnostic criteria for cognitive impairment or for diabetic retinopathy. | High risk. Diagnostic criteria for diabetic retinopathy is fine, but cut-off of a cognitive test was used to define mild cognitive impairment. |
| 1. Was the study instrument that measured the parameter of interest (i.e. age-related macular degeneration, glaucoma, diabetic retinopathy, cognitive impairment and dementia) shown to have reliability and validity? | Low risk. “Patients who were considered as having any sign of macular degeneration in color fundus photography were more closely evaluated, in order to be investigated, when necessary, with fluorescein angiography.” | Low risk. “Ophthalmic examination was performed and diagnosis was made by two glaucoma specialists.” | High risk. Diabetic retinopathy was not centrally adjudicated and it was not the main focus of this study. | Low risk. Funduscopic examination was carried out through dilated pupils by an ophthalmologist. |
| 1. Was the same mode of data collection used for all subjects? | Low risk. | Low risk. | Low risk. | Low risk. |
| 1. Were the numerator(s) and denominator(s) for the parameter of interest appropriate? | High risk. We need to calculate the prevalence rates and denominators. | Low risk. | Low risk. | High risk. The reported prevalence rate is incorrect. Typo? |

# eTable 4d Quality assessment

| Country/region, year, last name of the first author, study | Poland, 2014, 2016, Gorska-Ciebiada^56, 57^ | Singapore, 2013, Ong,^4^ Singapore Malay Eye Study | Spain, 2019, Marquié,^10^ NeuroOphthalmology Research At Fundació ACE cohort | Taiwan, 2015a, Chung^11^ |
| --- | --- | --- | --- | --- |
| 1. Was the study’s target population a close representation of the national population with age-related macular degeneration, glaucoma, diabetic retinopathy, cognitive impairment, mild cognitive impairment or dementia in relation to relevant variables, e.g. age, sex? | High risk. Multiple exclusion criteria making the target population not a close representation of the national population with diabetic retinopathy. | High risk. Aged 60 to 80 years who agreed to participate and underwent cognitive testing. | High risk. Excluded those patients with severe dementia stages, equivalent to a Global Deteriorating Scale score of >6. | High risk. Only included people ≥40 years old, diagnosed with dementia at least twice during the recruiting period with at least one diagnosis made by a certified neurologist, and excluded those with a history of major psychosis or a substance-related disorder prior to the first dementia diagnosis. |
| 1. Was the sampling frame a true or close representation of the target population? | High risk. Hospital-based. | Low risk. Population-based. | Low risk. Community-based. | Low risk. Population-based. |
| 1. Was some form of random selection used to select the sample, OR, was a census undertaken? | Low risk. ‘Unselected’. | Low risk. “All participants were given a choice to provide their written, informed consent” was mentioned in the cited previous publication. | Low risk. “Consecutive” was mentioned. | Low risk. All eligible cases included. |
| 1. Was the likelihood of non-response bias and/or lost to follow-up minimal? | High risk. No mention of response rate, and non-response was likely. | Low risk. Overall participation rate of 76.1%. The characteristics have been compared between those included and excluded and it has been reported that “Excluded persons were more likely to be older, to be diabetic, and to have lower education levels.” | High risk. Non-response rate was not reported. | Low risk. No need to consent. |
| 1. Were data collected directly from the subjects or medical records (as opposed to a proxy)? | Low risk. Directly from the subjects. | Low risk. All directly from the participants. | Low risk. Collected from the subjects. | Low risk. National Health Insurance collect data from hospitals, according to medical records. |
| 1. Was an acceptable case definition used in the study? | High risk. Diagnostic criteria for diabetic retinopathy unclear. | High risk. Cut off for the Abbreviated Mental Test was used to define cognitive decline. | Low risk. Diagnostic and Statistical Manual of Mental Disorders, fifth edition for dementia, Petersen`s criteria for mild cognitive impairment, and very detailed criteria for glaucoma and age-related macular degeneration. | Low risk. Both on the International Classification of Diseases, tenth revision codes. |
| 1. Was the study instrument that measured the parameter of interest (i.e. age-related macular degeneration, glaucoma, diabetic retinopathy, cognitive impairment and dementia) shown to have reliability and validity? | High risk. The diagnosis of diabetic retinopathy was not centrally adjudicated, and unsure about its validity. | High risk. “The education-specific cut points for the Abbreviated Mental Test have previously been validated against the Mini-Mental State Examination. For subjects with 0 to 6 years of education, the optimal cut point was 6 with a sensitivity of 89.6% and a specificity of 92.6%. For subjects with >6 years of education, the optimal cut point was 8 with a sensitivity of 82.1% and a specificity of 92.9%.” However, cut-off for Mini-Mental State Examination was not the a proper definition for cognitive decline, either. | Low risk. All assessments were carried out by a single optometrist and reviewed by a single ophthalmologist. | Low risk. “National Health Insurance Bureau of Taiwan maintains a regular cross-checking system with assessment and scrutiny of chart records from every hospital, followed by heavy penalties if discrepancies or instances of malpractice are discovered”, and “Previous studies that used the National Health Insurance Research Database demonstrated that it is of acceptable quality to provide reasonable estimates for epidemiological studies of neovascular age-related macular degeneration and dementia.” |
| 1. Was the same mode of data collection used for all subjects? | Low risk. | Low risk. | Low risk. | Low risk. |
| 1. Were the numerator(s) and denominator(s) for the parameter of interest appropriate? | Low risk. | High risk. The reported numerator, denominator and percentage did not match. | Low risk. | Low risk. |

# eTable 4e Quality assessment

| Country/region, year, last name of the first author, study | Taiwan, 2015b, Chung^25^ | Taiwan, 2017, Lai^26^ | UK, 2014, Keenan^18^ | UK, 2014, Williams^3^ |
| --- | --- | --- | --- | --- |
| 1. Was the study’s target population a close representation of the national population with age-related macular degeneration, glaucoma, diabetic retinopathy, cognitive impairment, mild cognitive impairment or dementia in relation to relevant variables, e.g. age, sex? | High risk. Only included those who received a first-time diagnosis of dementia during ambulatory care visits, or received a diagnosis of dementia at least twice coded in their ambulatory care claims, with at least one being made by a certified neurologist or psychiatrist. | High risk. People with glaucoma aged ≥65 and subjects who had the same diagnosis in three consecutive clinical records during the study period were included. | High risk. No specific exclusion criteria making the target population not representative, but in most cases patients undergoing admission for intravitreal anti-vascular endothelial growth factor therapy, neovascular age-related macular degeneration (rather than geographic atrophy or early age-related macular degeneration), similarly only dementia patients with an admission or day case care were included. | Low risk. Included people with Alzheimer’s disease aged ≥65, but not a problem considering the age onset of senile dementia. |
| 1. Was the sampling frame a true or close representation of the target population? | Low risk. Population-based. | Low risk. Population-based. | Low risk. Population-based. | High risk. Hospital-based. |
| 1. Was some form of random selection used to select the sample, OR, was a census undertaken? | Low risk. All eligible cases were included. | Low risk. All cases of glaucoma were included. | Low risk. Demographic, medical, and administrative information about all admissions to National Health Service hospitals in England and admissions funded by the National Health Service for treatment in non-National Health Service clinical organizations were used. | High risk. “The opportunistic approach for sampling was felt to offer the best chance of recruiting enough subjects while avoiding systematic bias, but a truly random approach would have used comprehensive AD patient database to sample cases.” |
| 1. Was the likelihood of non-response bias and/or lost to follow-up minimal? | Low risk. No need to consent. | Low risk. Consent was not required. | Low risk. Data linkage and no need to consent. | High risk. Response rate was not reported. |
| 1. Were data collected directly from the subjects or medical records (as opposed to a proxy)? | Low risk. National Health Insurance collect data from hospitals, according to medical records. | Low risk. “The database includes claim information, such as sex, date of birth, utilization of medical services, and diabetic retinopathies prescriptions” and when diagnoses were made at medical service, we assume those were directly collected from the participants. | Low risk. English national hospital episode statistics, assuming physicians examined the patients. | Low risk. “Alzheimer’s disease was defined by the National Institute of Neurological and Communicative Disorders and Stroke criteria applied by dementia specialists as part of routine clinical care”. In addition, “Dilated retinal photographs cantered on the macula were taken using a slit-lamp mounted Canon CR-DGi digital camera.” |
| 1. Was an acceptable case definition used in the study? | Low risk. Both on the International Classification of Diseases, 10th revision codes. | Low risk. Glaucoma and dementia diagnoses were both made on the International Classification of Diseases, ninth revision, clinical modification. | High risk. Diagnostic criteria not mentioned. | Low risk. The National Institute of Neurological and Communicative Diseases and Stroke/Alzheimer’s Disease and Related Disorders Association and age-related macular degeneration grading system (referred to in this study as the “Whitla grades”) grade definitions employed in the Rotterdam Study were used in this study. |
| 1. Was the study instrument that measured the parameter of interest (i.e. age-related macular degeneration, glaucoma, diabetic retinopathy, cognitive impairment and dementia) shown to have reliability and validity? | Low risk. “To preclude inaccurate medical claims, the National Health Insurance Bureau of Taiwan randomly samples a fixed percentage of claims data with review and scrutiny of chart records from every hospital, followed by heavy penalties if outlier practice or malpractice is discovered.”, although “open angle glaucoma and dementia diagnoses, which rely on administrative claims data and the International Classification of Diseases, 10th revision codes may be less precise than those made according to standardized diagnostic procedures including ophthalmologic and neuropsychological examinations.” | Low risk. “The diagnosis accuracy of these International Classification of Diseases, ninth revision, clinical modification codes has been completely evaluated in previous studies.” | High risk. No central adjudication or verification. | Low risk. Age-related macular degeneration grading was conducted by the study team. |
| 1. Was the same mode of data collection used for all subjects? | Low risk. | Low risk. | Low risk. | Low risk. |
| 1. Were the numerator(s) and denominator(s) for the parameter of interest appropriate? | Low risk. | Low risk. | High risk. Some errors in the reporting of the total number of participants. | High risk. Prevalence rate was not reported. |

# eTable 4f Quality assessment

| Country/region, year, last name of the first author, study | UK, 2015, Keenan^27^ | UK, 2016, Naidu^44^ | USA, 1978, Chandra^40^ | USA, 2019, Smilnak^14^ |
| --- | --- | --- | --- | --- |
| 1. Was the study’s target population a close representation of the national population with age-related macular degeneration, glaucoma, diabetic retinopathy, cognitive impairment, mild cognitive impairment or dementia in relation to relevant variables, e.g. age, sex? | High risk. No specific exclusion criteria making the target population not representative, but the authors commented that “this methodology will not capture all patients with primary open angle glaucoma in England, particularly those treated over a long period exclusively with medical therapy”, similarly only dementia patients with an admission or day case care were included. | High risk. Excluded those with diabetes other than type 2; those who were temporary residence and/or residence outside the catchment area; those who were lack of fluency in English; who moved from another primary care team; those who had a terminal or separate advanced condition; those who had severe mental illness; and those with severe advanced complications of diabetes. | High risk. People who had senile/presenile dementia as underlying, immediate, associated, or contributory cause of death in the United States of America in 1978, excluding people who were living with dementia. | High risk. Pathologic specimens of eyes and brains of autopsy subjects aged ≥75. |
| 1. Was the sampling frame a true or close representation of the target population? | Low risk. Population-based. | Low risk. Population-based. | Low risk. Population-based. | Low risk. Considering that target population were those who undergone autopsy, this hospital-based recruitment would frame a true or close representation of the target population. |
| 1. Was some form of random selection used to select the sample, OR, was a census undertaken? | Low risk. Demographic, medical, and administrative information about all admissions to National Health Service hospitals in England and admissions funded by the National Health Service for treatment in non-National Health Service clinical organizations were used. | Low risk. Of 1200 participants recruited at the time of the analyses carried out for this paper, 1084 (90.3%) had received cognitive assessment using the modified Telephone Interview for Cognitive Status (TICSM). Cases (cognitive impaired) were defined as participants with TICSM scores in the lowest 10% of the sample distribution (score ≤17). | Low risk. All dementia cases were included. | Low risk. All eligible cases were included. |
| 1. Was the likelihood of non-response bias and/or lost to follow-up minimal? | Low risk. Data linkage and no need to consent. | High risk. Diabetic retinopathy was defined as any retinal haemorrhage or microaneurysm found in either eye by an ophthalmologist, but not sure whether all the cognitively impaired cases were seen by an ophthalmologist. | Low risk. Consent was not required. | Low risk. “The need for participant consent was waived by the Institutional Review Board for this decedent research, as all research subjects are deceased, and all personal health information was used solely for research and was not disclosed to anyone outside Duke University without removing all identifiers.” |
| 1. Were data collected directly from the subjects or medical records (as opposed to a proxy)? | Low risk. English national hospital episode statistics, assuming physicians examined the patients. | Low risk. Collected from the subjects. | Low risk. We assume data from the death certificates were collected from the hospital medical records where the medical history were taken directly from the participants. | Low risk. Autopsy directly done on the participants, and demographic and clinical data collected from the electronic medical record and an online medical information system. |
| 1. Was an acceptable case definition used in the study? | Low risk. The International Classification of Diseases, 10th revision. | High risk. A cut-off was used for impaired cognition and the diagnostic criteria for diabetic retinopathy was unclear. | High risk. The diagnostic criteria for glaucoma was not mentioned. | Low risk. Braak and Braak (B&B) staging for Alzheimer’s disease, Sarks stages for age-related macular degeneration, and “the histopathologic diagnosis of advanced glaucoma was made when the following were observed: sparse retinal ganglion cells, diminished size of optic nerve axon bundles, and fibrotic thickening or “cupping” of the optic nerve.” |
| 1. Was the study instrument that measured the parameter of interest (i.e. age-related macular degeneration, glaucoma, diabetic retinopathy, cognitive impairment and dementia) shown to have reliability and validity? | High risk. No central adjudication or verification. | High risk. The diagnosis of diabetic retinopathy was not made by the study team. | High risk. Not sure about the validity of glaucoma diagnosis. | Low risk. Age-related macular degeneration severity was graded by a board-certified ophthalmic pathologist. Alzheimer’s disease was graded in brain specimens by neuropathologists. We would believe the glaucoma was graded by the ophthalmic pathologist. |
| 1. Was the same mode of data collection used for all subjects? | Low risk. | Low risk. | Low risk. | Low risk. |
| 1. Were the numerator(s) and denominator(s) for the parameter of interest appropriate? | High risk. Incident rate and person years of follow-up were not reported. | High risk. The numerator was not reported, and very likely not all those with impaired cognition had available information on whether they had diabetic retinopathy. | High risk. Prevalence rate was not reported. | High risk. The numerator for prevalence of glaucoma (number of glaucoma cases) was not reported, though all information was reported regarding the prevalence of age-related macular degeneration. |

# eTable 4g Quality assessment

| Country/region, year, last name of the first author, study | Australia, 2006, Pham,^5^ Blue Mountains Eye Study | Canada, 2015, Harrabi^16^ | China, 2020, Yu^45^ | Denmark, 2007, Kessing^28^ |
| --- | --- | --- | --- | --- |
| 1. Was the study’s target population a close representation of the national population with age-related macular degeneration, glaucoma, diabetic retinopathy, cognitive impairment, mild cognitive impairment or dementia in relation to relevant variables, e.g. age, sex? | High risk. To be included, participants must be aged ≥49, and participated in detailed examinations. | High risk. Very specific inclusion and exclusion criteria. Aged ≥65, for age-related macular degeneration cases bilateral age-related macular degeneration and visual acuity of worse than 20/40 in their better eye, for glaucoma cases bilateral glaucoma and a visual field mean deviation worse than or equal to 4dB in the worse eye, and excluded those having two or more of age-related macular degeneration, glaucoma and Fuch’s corneal dystrophy. | High risk. Excluded “acute inflammatory diseases and autoimmune disease, acute complications of diabetes mellitus, neurological diseases that cause cognitive impairment, other ocular conditions that affect visual function, patients with lung cancer, liver dysfunction, cardiac and respiratory failure, patients with alcoholism and drug abuse, and patients with incomplete information.” | Low risk. No specific exclusion criteria making the target population not representative. |
| 1. Was the sampling frame a true or close representation of the target population? | Low risk. Population-based | High risk. Hospital-based. | High risk. Hospital-based. | High risk. “In the study period from 1977 to 1995 only patients with the most severe forms of glaucoma were included in the study.” |
| 1. Was some form of random selection used to select the sample, OR, was a census undertaken? | Low risk. “A door-to-door census of two postcode areas west of Sydney, Australia” was mentioned in a cited publication. | Low risk. All eligible potential participants were invited to participate the study. | High risk. Unclear whether consecutive or random sampling. | Low risk. “Data on all patients admitted to all hospitals are collected routinely as a part of the official Danish health survey”. |
| 1. Was the likelihood of non-response bias and/or lost to follow-up minimal? | High risk. Although response rates ≥75.1%, not sure about the reason for declined participation, and the characteristics of those who were included and excluded were not reported. | High risk. 208(27%) refused plus 50(6%) were not capable of responding for themselves. Those who refused were 3 years older on average than those who participated and those who were unable to participate were 5 years older on average. Those who were unable to participate were more likely to be male than those who participated. | High risk. Non-response rate was not reported. | Low risk. Data linkage and no need to consent. |
| 1. Were data collected directly from the subjects or medical records (as opposed to a proxy)? | Low risk. “Data on demographic details, lifestyle factors and past medical histories were collected during an interview.” In addition, detailed examinations were conducted. | Low risk. Directly from the subjects. | Low risk. “Each subject was examined by a clinical ophthalmologist with the use of an ophthalmoscope. The diagnosis and staging of diabetic retinopathy were defined by fluorescein fundus angiography.” “All subjects were tested with the Mini-Mental State Examination scale.” | Low risk. Assuming physicians examined the patients. |
| 1. Was an acceptable case definition used in the study? | High risk. Cut off for Mini-Mental State Examination was used to define cognitive impairment, though the Wisconsin age-related macular degeneration Grading System was used to grade and define age-related macular degeneration. | High risk. Diagnostic criteria for age-related macular degeneration or glaucoma unclear. | High risk. The diagnostic criteria for diabetic retinopathy was unclear and cut-off on Mini-Mental State Examination was used for the diagnosis of mild cognitive impairment. | Low risk. International Classification of Diseases, eighth and 10th revision. |
| 1. Was the study instrument that measured the parameter of interest (i.e. age-related macular degeneration, glaucoma, diabetic retinopathy, cognitive impairment and dementia) shown to have reliability and validity? | High risk. The diagnostic performance of cut off of Mini-Mental State Examination to define cognitive impairment were not centrally adjudicated. | Low risk. Participants with scores <17 on Mini-Mental State Examination blind version meet the criteria for cognitive impairment. | Low risk. “All subjects were tested with the Mini-Mental State Examination scale.” | High risk. “The validity of diagnoses in the Danish National Hospital Register has been evaluated in prior studies, but the diagnoses of glaucoma, cataract, open-angle glaucoma, and dementia have not been specifically validated.” |
| 1. Was the same mode of data collection used for all subjects? | Low risk. | Low risk. | Low risk. | Low risk. |
| 1. Were the numerator(s) and denominator(s) for the parameter of interest appropriate? | Low risk. | High risk. The numerators were not reported. | High risk. The prevalence rate was not reported. | High risk. Incident rate and person-years of follow-up were not reported. |

# eTable 4h Quality assessment

| Country/region, year, last name of the first author, study | Denmark, 2012, Bach-Holm^23^ | Japan, 2017, Honjo^41^ | Malaysia, 2019, Raman^21^ | Netherlands, 1999, Klaver^2^ |
| --- | --- | --- | --- | --- |
| 1. Was the study’s target population a close representation of the national population with age-related macular degeneration, glaucoma, diabetic retinopathy, cognitive impairment, mild cognitive impairment or dementia in relation to relevant variables, e.g. age, sex? | High risk. Only patients with unilateral/bilateral normal tension glaucoma, making the target population not representative of the national population of people with glaucoma. | High risk. Target population aged ≥75, no history of dementia; axial length was between 26 mm and 21 mm; patients underwent at least three visual field tests, prior to the current study; and logMAR visual acuity ≤0.5, making the target population not representative of the national population of people with glaucoma. | High risk. Only included aged ≥50, best corrected visual acuity (6/12) in the better eye, no scotoma in the central 10 degree field of visual field, and excluded patients with ocular conditions, e.g. macular degeneration, media opacity, and dementia and other neurological diseases. | High risk. Only included those aged ≥75. |
| 1. Was the sampling frame a true or close representation of the target population? | High risk. Recruited from a specific hospital. | High risk. Hospital-based. | High risk. Hospital-based. | Low risk. Population-based. |
| 1. Was some form of random selection used to select the sample, OR, was a census undertaken? | Low risk. “All patients diagnosed with unilateral or bilateral normal tension glaucoma from January 1980 to December 2001 in the Glaucoma Clinic, University Hospital of Copenhagen (Rigshospitalet), Denmark, were included.” | Low risk. Not selective sampling. | Low risk. “The patients were randomly recruited from the glaucoma registry in the department of ophthalmology.” | Low risk. All eligible cases were included. |
| 1. Was the likelihood of non-response bias and/or lost to follow-up minimal? | Low risk. Since it was “all patients”, we assume no non-response bias. | High risk. Response rate not reported. | High risk. Non-response bias was not reported. | Low risk. 78% of those aged ≥55 agreed to participate in the baseline phase. |
| 1. Were data collected directly from the subjects or medical records (as opposed to a proxy)? | Low risk. Data from two nationwide registries, where hospital admissions to day or night care and psychiatric admissions have been recorded, assuming physicians examined all the patients. | Low risk. Data were collected from the subjects. | Low risk. “All the patients underwent complete ophthalmological examination including best corrected visual acuity, intraocular pressure measurement with Goldmann applanation tonometry, fundus photographs, and visual field test.” In addition, “cognitive assessment was carried out using clock drawing test”. | Low risk. Mainly from patients but in few cases from proxies or medical records. |
| 1. Was an acceptable case definition used in the study? | High risk. No standard diagnostic criteria used for normal tension glaucoma. | High risk. Mini-Mental State Examination score ≤23 defined as severe to moderate cognitive impairment; Mini-Mental State Examination score 24 to 27 points defined as mild cognitive impairment, and the diagnostic criteria for open angle glaucoma were not reported. | High risk. Cut off of the clock drawing test was used to define cognitive impairment. In addition, though “the Hodapp-Parrish-Anderson Glaucoma Grading Scale” was used to grade glaucoma severity, it was not clear the definition/diagnostic criteria for glaucoma. | Low risk. Alzheimer’s disease based on National Institute of Neurological and Communicative Diseases and Stroke/Alzheimer’s Disease and Related Disorders Association criteria |
| 1. Was the study instrument that measured the parameter of interest (i.e. age-related macular degeneration, glaucoma, diabetic retinopathy, cognitive impairment and dementia) shown to have reliability and validity? | High risk. Data from two nationwide registries, not sure about the validity. | High risk. The validity of mild cognitive impairment diagnosis not sure. | Low risk. A score of ≤3 is suggestive of cognitive impairment (83% sensitivity, 91% specificity) in a cited publication. | Low risk. Three steps approach. |
| 1. Was the same mode of data collection used for all subjects? | Low risk. | Low risk. | Low risk. | Low risk. |
| 1. Were the numerator(s) and denominator(s) for the parameter of interest appropriate? | High risk. Incidence rate was not reported. | High risk. The reported prevalence rates of cognitive impairment were calculated incorrectly. | High risk. The numerator was not reported. | High risk. Person-years of follow-up not reported. |

# eTable 4i Quality assessment

| Country/region, year, last name of the first author, study | Norway, 2004, Ritland^42^ | Singapore, 2012, Ong,^7^ Singapore Malay Eye Study | Singapore, 2019, Gupta^49^ | South Korea, 2012, Woo^8^ |
| --- | --- | --- | --- | --- |
| 1. Was the study’s target population a close representation of the national population with age-related macular degeneration, glaucoma, diabetic retinopathy, cognitive impairment, mild cognitive impairment or dementia in relation to relevant variables, e.g. age, sex? | High risk. Patients with diabetes mellitus were excluded. | Low risk. No specific exclusion criteria. | Low risk. Target population were adults aged 40 to 79 years living in Singapore, though in the analyses those without gradable fundus photos at baseline, etc, were excluded, which was counted in item 4 of this assessment tool. | High risk. Only included patients who had age-related macular degeneration categorized as ≥3 according to the Age-Related Eye Disease Study classification system. |
| 1. Was the sampling frame a true or close representation of the target population? | High risk. Hospital-based. | Low risk. Population-based. | Low-risk. Population-based. | High risk. Hospital-based. |
| 1. Was some form of random selection used to select the sample, OR, was a census undertaken? | Low risk. “Out of a total of 1320 registered patients, information on the lifetimes of 1147 individuals was available from the central population register of the Norwegian Government Computer Centre”. Very likely that all the eligible cases were included. | Low risk. All those eligible were invited to participate. | Low risk. All eligible cases were included. | Low risk. Randomly sampled. |
| 1. Was the likelihood of non-response bias and/or lost to follow-up minimal? | Low risk. No need to take consent. | High risk. Of the 2149 eligible persons aged 60 to 79 years, 1478 participated from 2004 to 2006. The overall participation rate was 76.1%. The response rates by age and sex were 76.5% and 77.2% in males aged 60 to 69 years and 70 to 79 years, respectively, and 78.0% and 72.0% in females aged 60 to 69 years and 70 to 79 years, respectively. Unclear how they differed. | High risk. Of those with diabetes (n = 1689), “1613 (95.5%) had gradable fundus photos at baseline” “741 attended the 6-year follow-up” and there was significant differences between those who were analysed and who were not. | High risk. Response rate was not reported. |
| 1. Were data collected directly from the subjects or medical records (as opposed to a proxy)? | Low risk. Data were collected from disease and death registry or from medical records, similar to from the patients directly. | Low risk. Collected from the subjects. | Low risk. Data were collected from the subjects. | Low risk. “We administered complete ophthalmic examinations, ...” and “Research neuropsychologists administered the Korean versions of the Consortium to Establish a Registry for Alzheimer’s Disease Neuropsychological Assessment Battery.” |
| 1. Was an acceptable case definition used in the study? | High risk. Diagnostic criteria for primary open angle glaucoma and for dementia were not reported. | High risk. Modified Airlie House Classification System was used for diabetic retinopathy, but cut-off on a scale was used for cognitive dysfunction. | Low risk. Cognitive impairment based on the score from Abbreviatd Mental Test ≤6 for education level between 0 to 6 years and ≤8 for education level greater than 6 years and Modified Airlie House classification system for diabetic retinopathy. | Low risk. Age-related macular degeneration categorized as ≥ 3 according to the Age-Related Eye Disease Study classification system and mild cognitive impairment was defined according to the Revised Diagnostic Criteria, proposed by the International Working Group on mild cognitive impairment. |
| 1. Was the study instrument that measured the parameter of interest (i.e. age-related macular degeneration, glaucoma, diabetic retinopathy, cognitive impairment and dementia) shown to have reliability and validity? | High risk. The diagnosis of dementia was not centrally adjudicated. | Low risk. The retinal photographs were assessed by the study team. | High risk. “The education-specific cut points for the Abbreviatd Mental Test have previously been validated against the Mini-Mental State Examination.” but the Mini-Mental State Examination was not the valid for dementia diagnosis. | Low risk. “Research neuropsychologists administered the Korean versions of the Consortium to Establish a Registry for Alzheimer’s Disease Neuropsychological Assessment Battery”. |
| 1. Was the same mode of data collection used for all subjects? | Low risk. | Low risk. | Low risk. | Low risk. |
| 1. Were the numerator(s) and denominator(s) for the parameter of interest appropriate? | High risk. Numerator/number of cases was not reported. | Low risk. | High risk. Person years of follow-up and incident rate were not reported. | High risk. The numerator (number of mild cognitive impairment cases) was not reported. |

# eTable 4j Quality assessment

| Country/region, year, last name of the first author, study | South Korea, 2018, Moon^37^ | South Korea, 2020, Choi^12^ | Taiwan, 2010, Lin^29^ | Taiwan, 2014, Lin^30^ |
| --- | --- | --- | --- | --- |
| 1. Was the study’s target population a close representation of the national population with age-related macular degeneration, glaucoma, diabetic retinopathy, cognitive impairment, mild cognitive impairment or dementia in relation to relevant variables, e.g. age, sex? | High risk. Participants must have been diagnosed with primary open-angle glaucoma (Korean Classification of Diseases code H401) as the main diagnosis code, having a visual field test code (E6691), and prescribed glaucoma medication. | High risk. Only included those aged ≥50. | High risk. At least three consensus open-angle glaucoma diagnoses, must receive anti-glaucoma medication or operations and must have undergone laser iridotomy or visual field examination. | High risk. Only included those aged ≥65, at least two visits with primary open angle glaucoma diagnosis over one-month apart, received anti-glaucoma medication or glaucoma surgery and visited at least once to an eye clinic or a hospital based ophthalmology department during the study period. |
| 1. Was the sampling frame a true or close representation of the target population? | Low risk. Population-based, “all citizens are obligated to enrol in the system.” | Low risk. Population-based. | Low risk. Population-based. | Low risk. Population-based. |
| 1. Was some form of random selection used to select the sample, OR, was a census undertaken? | Low risk. All eligible primary open angle glaucoma cases were included. | Low risk. All eligible cases were included. | Low risk. All eligible cases were included. | Low risk. All eligible cases were included. |
| 1. Was the likelihood of non-response bias and/or lost to follow-up minimal? | Low risk. “Need for written informed consent was waived”. | Low risk. No need to consent. | Low risk. No need to consent. | Low risk. No need to consent. |
| 1. Were data collected directly from the subjects or medical records (as opposed to a proxy)? | Low risk. Database contains personal demographic records to collect insurance premium and subscription data for reimbursement. | Low risk. “The Korean National Health Insurance Service provides mandatory health insurance to all citizens covering nearly all forms of healthcare services.” We assumed that services were delivered to subjects directly hence the data were collected from the subjects directly. | Low risk. National Health Insurance collect data from hospitals, sourced from the medical records. | Low risk. National Health Insurance collected data from hospitals. Data were sourced from the medical records. |
| 1. Was an acceptable case definition used in the study? | Low risk. Both used the Korean Classification of Diseases codes. | Low risk. Coded as International Classification of Diseases, tenth revision codes, and “Operational definitions for age-related macular degeneration, and Alzheimer`s disease were adopted from previous studies.” | Low risk. International Classification of Diseases, ninth revision, clinical modification codes. | Low risk. International Classification of Diseases, ninth revision. |
| 1. Was the study instrument that measured the parameter of interest (i.e. age-related macular degeneration, glaucoma, diabetic retinopathy, cognitive impairment and dementia) shown to have reliability and validity? | High risk. The diagnosis was not centrally adjudicated. | High risk. The authors “did not have access to medical chart records, the definitions of age-related macular degeneration, and Alzheimer`s disease could not be verified with certainty.” | Low risk. “National Health Insurance Bureau of Taiwan randomly samples a fixed percentage of claims from every hospital and randomly interviews patients and reviews charts each year to verify the diagnosis validity and quality of care. Any hospital with outlier charges or outlier practice patterns or that is suspected of malpractice faces the risk of an audit and subsequent heavy penalties.” | Low risk. “To ensure the validity of disease diagnoses, we assigned diagnoses based on claims for two visits at least 30 days apart with the same diagnosis code, as well as related prescription claims.” |
| 1. Was the same mode of data collection used for all subjects? | Low risk. | Low risk. | Low risk. | Low risk. |
| 1. Were the numerator(s) and denominator(s) for the parameter of interest appropriate? | High risk. Person-years of follow-up was not reported. | High risk. Incidence rate was not reported. | Low risk. | Low risk. |

# eTable 4k Quality assessment

| Country/region, year, last name of the first author, study | Taiwan, 2015, Tsai^13^ | Taiwan, 2016, Su^31^ | Taiwan, 2017, Lai^33^ | Taiwan, 2018, Chen^32^ |
| --- | --- | --- | --- | --- |
| 1. Was the study’s target population a close representation of the national population with age-related macular degeneration, glaucoma, diabetic retinopathy, cognitive impairment, mild cognitive impairment or dementia in relation to relevant variables, e.g. age, sex? | High risk. Included those “aged ≥65 years and had ≥2 clinical visits with a diagnosis of age-related macular degeneration, excluded those who was diagnosed with age-related macular degeneration before 2001, diagnosed as Alzheimer’s disease or dementia before enrolment, and a history of stroke. | Low risk. Newly diagnosed with glaucoma during the recruiting period. | High risk. Only one dementia type Alzheimer’s disease and only included those who were ≥65 years old. | Low risk. No specific exclusion criteria. |
| 1. Was the sampling frame a true or close representation of the target population? | Low risk. Population-based. | Low risk. Population-based. | Low risk. Population-based, “the insurance program began in March 1995 and has covered 99% of the entire population of 23 million people living in Taiwan.” | Low risk. Population-based. |
| 1. Was some form of random selection used to select the sample, OR, was a census undertaken? | Low risk. Very likely all the eligible cases were included. | Low risk. Included all eligible cases. | Low risk. All cases with Alzheimer’s disease were included. | Low risk. All eligible cases were included. |
| 1. Was the likelihood of non-response bias and/or lost to follow-up minimal? | Low risk. No need to consent. | Low risk. No need to consent, as the original identification numbers of insurants have been moved to protect their confidentiality. | Low risk. Consent was not required. | Low risk. Consent was not required. |
| 1. Were data collected directly from the subjects or medical records (as opposed to a proxy)? | Low risk. The database contains the registry for beneficiaries, outpatient and inpatient files, drug prescriptions registry, and data on other medical services availed by the insurants. | Low risk. The database contains the registry for beneficiaries, outpatient and inpatient files, drug prescriptions registry, and data on other medical services availed by the insurants. | Low risk. “The database includes claim information, such as sex, date of birth, utilization of medical services, and diabetic retinopathies prescriptions” and when diagnoses were made at medical service, we assume those were directly collected from the participants. | Low risk. National Health Insurance collect data from hospitals, sourced from the medical records. |
| 1. Was an acceptable case definition used in the study? | Low risk. Both on the International Classification of Diseases, ninth revision, clinical modification. | Low risk. International Classification of Diseases, ninth revision, clinical modification, used to code glaucoma and dementia. | Low risk. Coded using International Classification of Diseases, ninth revision, clinical modification, though as mentioned by the authors “we could not know what the criterion for diagnosing glaucoma was”. “Low risk” is to be consistent with our judgements for all the studies using the International Classification of Diseases clinical modification. | Low risk. Glaucoma and dementia diagnoses were both made on the International Classification of Diseases, ninth revision, clinical modification. |
| 1. Was the study instrument that measured the parameter of interest (i.e. age-related macular degeneration, glaucoma, diabetic retinopathy, cognitive impairment and dementia) shown to have reliability and validity? | High risk. Although “To improve the identification accuracy, those who received an Alzheimer’s disease or senile dementia diagnosis only once were not identified as incident Alzheimer’s disease or senile dementia cases.”, we are unsure about the validity of the recorded diagnosis of Alzheimer’s disease. | High risk. No centrally validation or adjudication. | Low risk. “The diagnosis accuracy based on International Classification of Diseases, ninth revision, clinical modification codes has been completely examined in previous studies”. | High risk. “The diagnosis of Alzheimer’s disease was confirmed by neurologists or psychiatrists through a well-acknowledged, standard diagnostic protocol.” but it is unclear what the protocol was. |
| 1. Was the same mode of data collection used for all subjects? | Low risk. | Low risk. | Low risk. | Low risk. |
| 1. Were the numerator(s) and denominator(s) for the parameter of interest appropriate? | High risk. Did not report person-years of follow-up or incidence rate. | Low risk. | Low risk. | High risk. Person-years of follow-up and incidence rate were not reported, only reported accumulative incidence rate. |

# eTable 4l Quality assessment

| Country/region, year, last name of the first author, study | Taiwan, 2019, Chen^34^ | Taiwan, 2020, Kuo^35^ | Turkey, 2014, Dag^17^ | Turkey, 2015, Seden^9^ |
| --- | --- | --- | --- | --- |
| 1. Was the study’s target population a close representation of the national population with age-related macular degeneration, glaucoma, diabetic retinopathy, cognitive impairment, mild cognitive impairment or dementia in relation to relevant variables, e.g. age, sex? | High risk. Patients with a history of primary open angle glaucoma diagnosed before date of diagnosis of primary angle close glaucoma and/or with juvenile and congenital glaucoma were excluded. | High risk. Exclude those with blindness, an ophthalmic malignancy, eyeball removal surgery, and severe ocular trauma. | High risk. Included those aged ≥60, with bilateral age-related macular degeneration, and best corrected visual acuity no worse than 2/10, and excluded those with glaucoma, diabetic retinopathy, or cataract surgery within 6 months, dementia, and life threatening illness. | High risk. Aged ≥65, no Alzheimer’s disease, and sufficient visual acuity (≥20/100), especially it was not necessary to exclude people with previous diagnosis of Alzheimer’s disease. |
| 1. Was the sampling frame a true or close representation of the target population? | Low risk. Population-based. | Low risk. Population-based. | High risk. Single centre hospital-based. | High risk. Hospital-based. |
| 1. Was some form of random selection used to select the sample, OR, was a census undertaken? | Low risk. All eligible cases were included. | Low risk. All eligible cases were included. | High risk. Not sure whether the study sample was a subsample of an existing study, etc. Not sure whether consecutive sampling. | Low risk. All the eligible cases were included. |
| 1. Was the likelihood of non-response bias and/or lost to follow-up minimal? | Low risk. No need to consent. | Low risk. No need to consent. | High risk. The response rate was not reported. | High risk. The response rate was not reported. |
| 1. Were data collected directly from the subjects or medical records (as opposed to a proxy)? | Low risk. National Health Insurance collected data from hospitals. Data were sourced from the medical records. | Low risk. Medical records. | Low risk. Cognitive assessments (Mini-Mental State Examination and the Montreal Cognitive Assessment) were conducted directly with the participants, age-related macular degeneration participants were identified from the hospital and should have been checked directly by the study team. | Low risk. “All patients were questioned for basic information, including demographics, presence of concomitant diseases, drug usage, and exposure to potential risk factors for age-related macular degeneration or Alzheimer’s disease. Following that, all patients and controls underwent detailed ophthalmological, neurological, and psychiatric examinations on the same day.” |
| 1. Was an acceptable case definition used in the study? | Low risk. International Classification of Diseases, ninth revision. | High risk. Glaucoma diagnostic criteria not reported (though could be the International Classification of Diseases, the ninth and 10th revision. | High risk. Cut-offs of the Mini-Mental State Examination and the Montreal Cognitive Assessment were used to define mild cognitive impairment, and age-related macular degeneration definition was not reported. | Low risk. Age-related macular degeneration based on Age-Related Eye Disease Study, and Alzheimer’s disease based on the National Institute of Neurological and Communicative Disorders and Stroke and the Alzheimer’s Disease and Related Disorders Association. |
| 1. Was the study instrument that measured the parameter of interest (i.e. age-related macular degeneration, glaucoma, diabetic retinopathy, cognitive impairment and dementia) shown to have reliability and validity? | High risk. No related reports. | High risk. Validity not reported. | Low risk. Cognitive assessments (Mini-Mental State Examination and the Montreal Cognitive Assessment) were conducted directly with the participants by the researchers. | Low risk. All underwent detailed ophthalmological, neurological, and psychiatric examinations. |
| 1. Was the same mode of data collection used for all subjects? | Low risk. | Low risk. | Low risk. | Low risk. |
| 1. Were the numerator(s) and denominator(s) for the parameter of interest appropriate? | High risk. Prevalence rate was not reported. | Low risk. Note that the incident rates were reported based on person-months and we re-calculated to person-years. | Low risk. | Low risk. |

# eTable 4m Quality assessment

| Country/region, year, last name of the first author, study | Turkey, 2015, Ogurel^46^ | UK, 2013, Crosby-Nwaobi,^50^ The South East London Diabetic Retinopathy Study | USA, 2009, Baker,^6^ The Cardiovascular Health Study | USA, 2011, Whitson^19^ |
| --- | --- | --- | --- | --- |
| 1. Was the study’s target population a close representation of the national population with age-related macular degeneration, glaucoma, diabetic retinopathy, cognitive impairment, mild cognitive impairment or dementia in relation to relevant variables, e.g. age, sex? | High risk. Excluded those with history of ocular trauma, ocular surgery within 6 months, best corrected visual acuity worse than 1/10, history of cerebrovascular events, the presence of life threatening illness and dementia. | High risk. Twenty patients with a severe mental illness (the majority had a psychotic disorder), 20 with dementia and 25 with a history of stroke in the proliferative diabetic retinopathy group were excluded. Patients with a best corrected visual acuity ≤6/60 were excluded because they would not have been able to perform a number of the cognitive impairment measures. Their exclusion means that patients with the most extensive diabetic retinopathy were not included in the analysis and that these patients may have had an elevated risk of cognitive impairment. | High risk. Only early age-related macular degeneration was included and many specified inclusion and exclusion criteria, making the target population not representative of the national population with age-related macular degeneration. | High risk. Included those aged ≥65 with macular disease and excluded those with hearing problem and/or language barriers. |
| 1. Was the sampling frame a true or close representation of the target population? | High risk. Not clear where the participants were recruited from, but very likely from hospital settings. | Low risk. Community-based. | Low risk. This is a population-based study. | High risk. Hospital-based. |
| 1. Was some form of random selection used to select the sample, OR, was a census undertaken? | Low risk. “Consecutive” was mentioned. | Low risk. All those eligible and consented were included. | Low risk. “In 1992 to 1993, an additional 687 black individuals were recruited into the study from three sites”, which might have been questionable, but considering that it is unclear whether these black individuals were more likely to have early age-related macular degeneration and over-represented, this may be less of a problem. | Low risk. This study “invited all eligible patients until the weekly recruitment goal of three to five patients was met”. Although this is not consecutive, this is random sampling. |
| 1. Was the likelihood of non-response bias and/or lost to follow-up minimal? | High risk. Not sure about the non-response rate. | High risk. “Of 581 eligible persons approached, 380 agreed to participate (65.4%). Nonparticipants were older (mean age 68.6 ±11 versus 65.6 ±11 years) and had poorer visual acuity (mean logMAR 0.18 ± 0.3 versus 0.12±0.2) than participants.” | High risk. As mentioned in the study “of the 707 participants evaluated as having dementia in the Cardiovascular Health Study, only 145 (20.5%) had a gradable retinal photograph and could be assessed for age-related macular degeneration.” “Moderate to severe dementia generally hampers the performance of clinical investigations such as retinal photography.” | Low risk. “Of 139 patients invited to participate, 103 (74.1%) signed consent forms and 101 received cognitive testing at baseline. Those who declined to participate did not differ significantly from study participants on the basis of sex, race, or age.” |
| 1. Were data collected directly from the subjects or medical records (as opposed to a proxy)? | Low risk. Collected from the subjects. | Low risk. “Data were collected during a standardized clinical assessment and notes review undertaken by a researcher.” | Low risk. Data were collected directly from the participants. | Low risk. “Data were collected at baseline and during three follow-up telephone interviews.” |
| 1. Was an acceptable case definition used in the study? | High risk. Not a proper diagnostic criteria for cognitive impairment. | High risk. Cut-off points of Mini-Mental State Examination or Mini-Cog were used to define cognitive impairment and dementia. | Low risk. Dementia (Diagnostic and Statistical Manual of Mental Disorders, fourth edition), Alzheimer’s disease (National Institute of Neurological and Communicative Diseases and Stroke/Alzheimer’s Disease and Related Disorders Association), age-related macular degeneration (Modified Wisconsin age-related macular degeneration grading system), though lowest quartile of the distribution of scores (Digit Symbol Substitution Test ≤30; Modified Mini-Mental State Examination ≤89) was not acceptable, these were termed as “low cognitive function or mild cognitive impairment”. | High risk. Diagnostic criteria for wet and dry age-related macular degeneration unclear and cut off on the Telephone Interview for Cognitive Status (modified) was used. |
| 1. Was the study instrument that measured the parameter of interest (i.e. age-related macular degeneration, glaucoma, diabetic retinopathy, cognitive impairment and dementia) shown to have reliability and validity? | Low risk. The Montreal Cognitive Assessment and Mini-Mental State Exam were administered by the study investigators. | Low risk. Mini-Mental State Examination and Mini-Cog were assessed by the study team with the subjects directly. | Low risk. It was mentioned in an earlier report that “evaluated for dementia by detailed neurological, and neuropsychological examinations. The possible cases of dementia and mild cognitive impairment were adjudicated by a review committee of neurologists and psychiatrists.” | Low risk. Telephone Interview for Cognitive Status (modified) was “administered in person, in private exam rooms by trained administrators under supervision by a neuropsychologist.” |
| 1. Was the same mode of data collection used for all subjects? | Low risk. | Low risk. | Low risk. | Low risk. |
| 1. Were the numerator(s) and denominator(s) for the parameter of interest appropriate? | Low risk. | Low risk. | High risk. Denominators and prevalence rates were not given and we calculated them. | High risk. The numerator, denominator and prevalence rates were not reported. |

# eTable 4n Quality assessment

| Country/region, year, last name of the first author, study | USA, 2012, Ou^36^ | USA, 2014, Exalto^51^ | USA, 2018, Rodill^52^ | USA, 2020, Schwaber^15^ |
| --- | --- | --- | --- | --- |
| 1. Was the study’s target population a close representation of the national population with age-related macular degeneration, glaucoma, diabetic retinopathy, cognitive impairment, mild cognitive impairment or dementia in relation to relevant variables, e.g. age, sex? | High risk. Only included those who were aged ≥68, and had a diagnosis of open-angle glaucoma at two claims or a combined of one claim for open-angle glaucoma diagnosis and one claim for open-angle glaucoma related procedure. Participants also had to be present in the system at least another year after January 2014 and had at least three visits for healthcare during the follow-up period. | High risk. To be included, participants were required to have no gap of ≥3 months in health plan membership during the two years prior to baseline, aged ≥60 years at baseline. To restrict false positive diagnosis of diabetic retinal disease, only the severe sight threatening diabetic retinal disease forms (proliferative diabetic retinopathy and diabetic macular edema) were included. | High risk. Patients were considered if they fit all three of the following criteria: a) had either two International Statistical Classification of Diseases, ninth edition, codes for type 1 diabetes without any type 2 diabetes codes, or a ratio of type 1:type 2 diabetes International Statistical Classification of Diseases, ninth edition, codes higher than or equal to 0.75 in the Kaiser Permanente electronic database since start of membership; b) filled insulin prescriptions during the study period; and c) did not have filled prescriptions of any hypoglycemic agent other than insulin or metformin. | High risk. Autopsy cases, aged >75. |
| 1. Was the sampling frame a true or close representation of the target population? | Low risk. Population-based. | Low risk. Population-based. | Low risk. Population-based and “Senior (65+) members of Kaiser Permanente Northern California are similar to seniors residing in the region with respect to history of chronic conditions, including diabetes, hypertension, heart disease and lifestyle factors (e.g. smoking, a sedentary lifestyle and obesity).” | Low risk. Considering that target population were those who undergone autopsy, this hospital-based recruitment would frame a true or close representation of the target population. |
| 1. Was some form of random selection used to select the sample, OR, was a census undertaken? | Low risk. 5% of the medicare population was selected. | Low risk. “All” members of the Registry were mailed a survey to collect sociodemographic and health behaviours information. | Low risk. “Members of Kaiser Permanente Northern California with type 1 diabetes, with no prevalent dementia diagnoses, and at least 50 years old at any time within the study period (01/01/1996 until 9/30/2015) were eligible for our dynamic cohort.” | Low risk. All eligible were included. |
| 1. Was the likelihood of non-response bias and/or lost to follow-up minimal? | Low risk. No need to consent. | Low risk. Response rate was 83%, and “Previously, we found no indication of bias from differences in characteristics or epidemiologic associations by survey respondent status.” | Low risk. No need to consent. | Low risk. No need to consent. |
| 1. Were data collected directly from the subjects or medical records (as opposed to a proxy)? | Low risk. Medicare claims data, sourced from medical records. | Low risk. Data were collected using data from the survey and the combined electronic medical record data which incorporates laboratory test results, pharmacy, inpatient and outpatient diagnoses. | Low risk. Kaiser Permanente Northern California database collected data from “primary care, neurology, or memory clinic visits”, similar to medical records which were collected directly from the participants. | Low risk. Autopsy directly done on the participants, and demographic and clinical data collected from the electronic medical record and an online medical information system. |
| 1. Was an acceptable case definition used in the study? | Low risk. Both on International Classification of Diseases, ninth revision, clinical modification. | Low risk. Both on the International Classification of Diseases, ninth revision, clinical modification. | Low risk. International Statistical Classification of Diseases, ninth edition, codes were used for both. | Low risk. Sarks stages for age-related macular degeneration, and Braak and Braak (B&B) staging for Alzheimer’s Disease |
| 1. Was the study instrument that measured the parameter of interest (i.e. age-related macular degeneration, glaucoma, diabetic retinopathy, cognitive impairment and dementia) shown to have reliability and validity? | High risk. The diagnosis of Alzheimer’s disease or other dementia was not centrally adjudicated. | Low risk. “This strategy was found to have a sensitivity of 77% and specificity of 95% compared to a consensus diagnosis of dementia based on a neuropsychiatric battery, physical examination, structured interview with informants, and review of medical records.” | Low risk. “A similar combination of International Statistical Classification of Diseases, ninth edition, codes had a sensitivity of 77% and a specificity of 95% compared to a consensus diagnosis of dementia based on a structured interview with informants, neuropsychiatric battery, physical examination and review of medical records.” | Low risk. “All relevant neuropathological and ophthalmic diagnoses were verified through recorded immunohistochemistry and silver stains that were reviewed by a senior ocular pathologist.” |
| 1. Was the same mode of data collection used for all subjects? | Low risk. | Low risk. | Low risk. | Low risk. |
| 1. Were the numerator(s) and denominator(s) for the parameter of interest appropriate? | High risk. Person-years of follow-up and incidence rates were not reported. | Low risk. | High risk. Person-years of follow up and incidence rate were not reported. | Low risk. |
